# Gata6 potently initiates reprograming of pluripotent and differentiated cells to extraembryonic endoderm stem cells

**DOI:** 10.1101/gad.257071.114

**Published:** 2015-06-15

**Authors:** Sissy E. Wamaitha, Ignacio del Valle, Lily T.Y. Cho, Yingying Wei, Norah M.E. Fogarty, Paul Blakeley, Richard I. Sherwood, Hongkai Ji, Kathy K. Niakan

**Affiliations:** 1 Mill Hill Laboratory, The Francis Crick Institute, London NW7 1AA, United Kingdom;; 2 Department of Biostatistics, Bloomberg School of Public Health, Johns Hopkins University, Baltimore, Maryland 21205, USA;; 3 Brigham and Women's Department of Medicine, Harvard Medical School, Boston, Massachusetts 02115, USA

**Keywords:** reprograming, pluripotency, mouse embryonic stem cells, human embryonic stem cells, extraembryonic endoderm, Gata6

## Abstract

Transcription factor-mediated reprograming is a powerful method to study cell fate changes. In this study, we demonstrate that the transcription factor Gata6 can initiate reprograming of multiple cell types to induced extraembryonic endoderm stem (iXEN) cells. Intriguingly, Gata6 is sufficient to drive iXEN cells from mouse pluripotent cells and differentiated neural cells. Furthermore, GATA6 induction in human embryonic stem (hES) cells also down-regulates pluripotency gene expression and up-regulates extraembryonic endoderm (ExEn) genes, revealing a conserved function in mediating this cell fate switch. Profiling transcriptional changes following Gata6 induction in mES cells reveals step-wise pluripotency factor disengagement, with initial repression of *Nanog* and *Esrrb*, then *Sox2*, and finally *Oct4*, alongside step-wise activation of ExEn genes. Chromatin immunoprecipitation and subsequent high-throughput sequencing analysis shows Gata6 enrichment near pluripotency and endoderm genes, suggesting that Gata6 functions as both a direct repressor and activator. Together, this demonstrates that Gata6 is a versatile and potent reprograming factor that can act alone to drive a cell fate switch from diverse cell types.

## Abstract (precis)

Wamaitha et al. demonstrate that the transcription factor Gata6 can initiate reprograming of multiple cell types to induced extraembryonic endoderm cells. Profiling transcriptional changes following Gata6 induction in mES cells reveals step-wise pluripotency factor disengagement, with initial repression of Nanog and Esrrb, then Sox2, and finally Oct4, alongside step-wise activation of extraembryonic endoderm genes.

One of the earliest specification events during mammalian development is the divergence of pluripotent epiblast progenitor (EPI) cells, which give rise to the embryo proper, and primitive endoderm (PrE) cells, which mainly contribute to the yolk sac. In mice, embryonic stem (ES) cells and extraembryonic endoderm stem (XEN) cells can be derived from the EPI and PrE lineages, respectively, and retain characteristics of their cell type of origin (Evans and Kaufman 1981; Martin 1981; Kunath et al. 2005). Mouse ES (mES) cells are able to self-renew and remain pluripotent and depend on a gene regulatory network surrounding the core transcription factors Oct4, Sox2, and Nanog (Boyer et al. 2005; Loh et al. 2006; Chen et al. 2008). Transcription factors such as Esrrb and Klf4 are also regulated by and reinforce the core pluripotency factors (Ivanova et al. 2006; van den Berg et al. 2008; Zhang et al. 2008; Hall et al. 2009; Niwa et al. 2009; Festuccia et al. 2012).

In contrast, PrE and self-renewing XEN cells lack expression of pluripotency genes and require an extraembryonic endoderm (ExEn) program, including transcription factors Gata4, Gata6, Sox7, and Sox17 (Soudais et al. 1995; Arman et al. 1998; Morrisey et al. 1998; Koutsourakis et al. 1999; Futaki et al. 2004; Capo-Chichi et al. 2005; Kunath et al. 2005; Chazaud et al. 2006; Shimoda et al. 2007; Brown et al. 2010b; Morris et al. 2010; Niakan et al. 2010; Artus et al. 2011; Schrode et al. 2014). It remains unclear how cells within the early embryo diverge to favor either a pluripotency or an ExEn gene regulatory network. The overexpression of the zinc finger transcription factor Gata6 or Gata4 is sufficient to reprogram mES cells into XEN-like cells that contribute to PrE-derived lineages in vivo (Fujikura et al. 2002; Shimosato et al. 2007). The GATA factors are therefore part of a subset of transcription factors that are capable of inducing cellular reprograming, although precisely how this occurs has yet to be elucidated. Ectopic expression of the transcription factor MyoD converts fibroblasts to myogenic cells (Davis et al. 1987), a combination of transcription factors reprograms fibroblasts to induced pluripotent stem (iPS) cells (Takahashi and Yamanaka 2006), and in vivo pancreatic exocrine cells can be reprogramed into insulin-secreting β-like cells (Zhou et al. 2008). Similarly, overexpression of Cdx2 is sufficient to reprogram mES cells to trophectoderm-like cells that contribute solely to placental lineages in vivo (Niwa et al. 2005). However, the mechanisms by which single transcription factors reprogram existing gene expression patterns toward that of their target cell type remain unclear.

Induction of the SRY homeobox gene *Sox17* has also been shown to induce ExEn gene expression in mES cells (Niakan et al. 2010; McDonald et al. 2014). Intriguingly, *SOX17* induction in human ES (hES) cells instead drives an embryonic endoderm program (Seguin et al. 2008). This incongruence is consistent with our previous observations that the initial ES cell state influences differentiation outcomes (Cho et al. 2012). Furthermore, while the induction of *SOX7* drives ExEn gene expression in hES cells (Seguin et al. 2008), stable self-renewing human XEN cells have yet to be established. The effect of GATA factor induction in hES cells has not been tested, and it is unclear whether *Gata6* can function as a master transcriptional regulator to induce a XEN program from cells other than mES cells.

We developed a highly efficient approach to understand the molecular mechanisms of Gata6-mediated reprograming and show that Gata6 is a potent inducer of lineage reprograming in multiple cell types. We demonstrate that a short pulse of *Gata6* induction is ample to perturb gene expression in mES cells and initiate conversion to induced XEN (iXEN) cells, while longer induction fully down-regulates the pluripotency program. Using genome-wide transcriptional and chromatin immunoprecipitation (ChIP) analyses, we found that Gata6 is able to rapidly and directly inhibit core and peripheral genes within the pluripotency regulatory network as well as directly activate an ExEn program. Despite lingering expression of Oct4 following *Gata6* induction, loss-of-function analysis suggests that Oct4 is not required to drive this lineage switch in mES cells. Gata6 expression in more committed neural cells also drives reprograming to iXEN-like cells. We show that *GATA6* induction in hES cells initiates ExEn expression and is sufficient to inhibit core pluripotency gene expression. Our findings have important implications for understanding how transcription factors function to drive a cell fate switch and provide fundamental insights into early mammalian cell fate specification.

## Results

### *Gata6* or *Gata4* expression is uniquely sufficient to induce rapid reprograming of mES cells to iXEN cells

While Gata4 and Gata6 are able to reprogram mES cells, it is unclear whether other endoderm transcription factors are also able to mediate this cell fate switch. We selected six transcription factors (Gata4, Gata6, Hnf4a, Foxa3, Sox7, and Sox17) that are expressed in the PrE or its derivatives and are functionally required to establish or maintain this lineage (Chen et al. 1994; Soudais et al. 1995; Molkentin et al. 1997; Kaestner et al. 1998; Morrisey et al. 1998; Koutsourakis et al. 1999; Capo-Chichi et al. 2005; Artus et al. 2011; Schrode et al. 2014). To investigate whether their expression is sufficient to induce reprograming of mES cells to iXEN cells, we used a site-specific recombination-based integration strategy (Hochedlinger et al. 2005; Beard et al. 2006) to generate mES cells expressing a single copy of a tetracycline/doxycycline-inducible *Gata4*, *Gata6*, *Sox7*, *Sox17*, *Hnf4a*, or *Foxa3* transgene. To test the fidelity of the system, we also engineered control mES cells that induce the expression of a gene encoding a red fluorescent protein, *dsRed*. We confirmed robust induction above or close to levels present in embryo-derived XEN (eXEN) cells by quantitative RT–PCR (qRT–PCR) analysis of the Flag- or V5-tagged transgene (Supplemental Fig. S1A). We also observed robust red fluorescence in *dsRed*-overexpressing mES cells (Supplemental Fig. S1B).

**Figure 1.**
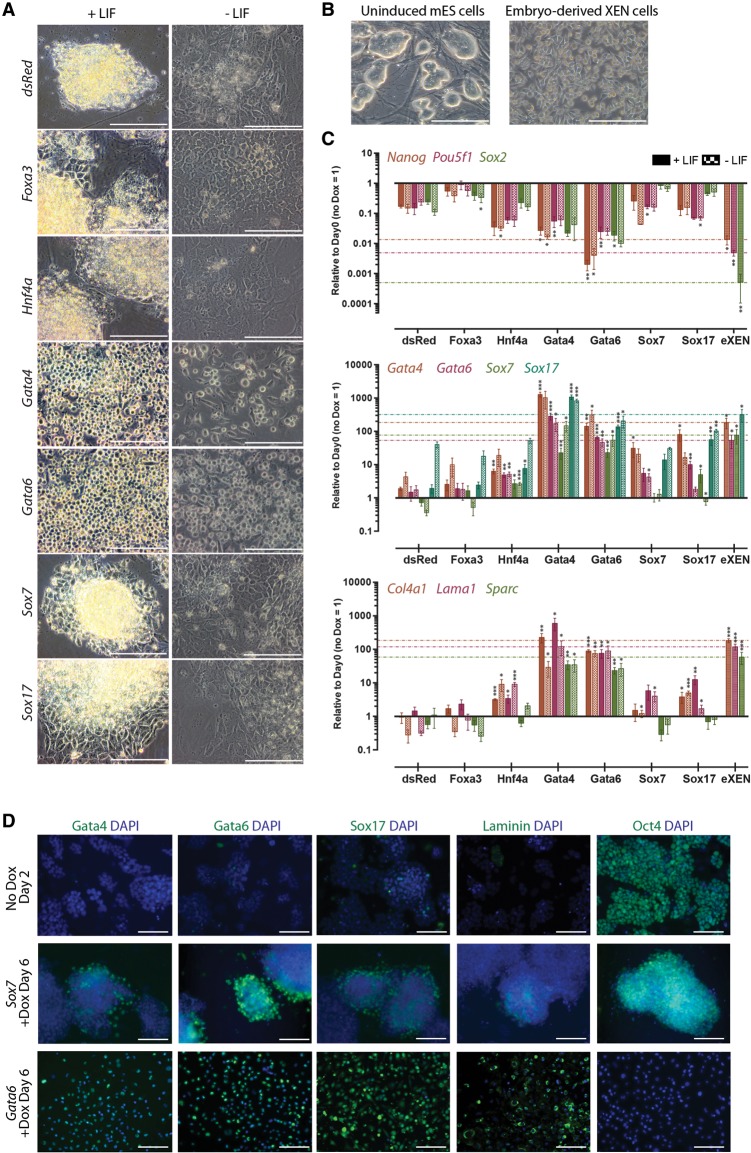
*Gata6* or *Gata4* induction is uniquely sufficient to reprogram mES cells to XEN cells. (*A*) Representative phase-contrast images of *dsRed*-, *Foxa3*-, *Hnf4a*-, *Gata4*-, *Gata6*-, *Sox7*-, or *Sox17*-induced cells after 6 d of doxycycline treatment in the presence or absence of LIF. Bars, 100 μm. (*B*) Phase-contrast images of uninduced mES cells and eXEN cells. (*C*) qRT–PCR analysis for selected pluripotency and endoderm transcripts in *dsRed*-, *Foxa3*-, *Hnf4a*-, *Gata4*-, *Gata6*-, *Sox7*-, or *Sox17*-induced cells after 6 d of doxycycline treatment in the presence (solid) or absence (checkered) of LIF. Relative expression reflected as fold difference over uninduced mES cells normalized to *Gapdh*. Data are mean ± SEM of two to three biological replicates and four technical replicates. (*) *P* < 0.05; (**) *P* < 0.01; (***) *P* <0 .001. (*D*) Immunofluorescence analysis for Gata4, Gata6, Sox17, Laminin, or Oct4 (all green) with DAPI merge (blue) in uninduced mES cells or *Sox7*- or *Gata6*-overexpressing mES cells after 6 d of doxycycline treatment.

To determine the effect of these factors under conditions that would otherwise maintain pluripotency, we induced exogenous expression with doxycycline for 6 d in the presence of LIF and serum, which are known to maintain mES cell self-renewal indefinitely in culture (Fig. 1A). *Gata6* or *Gata4* overexpression resulted in reprograming to cells with the dispersed, refractile, and stellate morphology characteristic of eXEN (Fig. 1B) and growth factor-converted XEN (cXEN) cells (Kunath et al. 2005; Cho et al. 2012). qRT–PCR analysis of the 3′ untranslated region (UTR) confirmed that *Gata6*- or *Gata4*-induced cells expressed endogenous *Gata6* and *Gata4* as well as key ExEn genes, including *Sox7*, *Sox17*, *Lama1*, *Col4a1*, *Sparc*, *Dab2*, *Foxa3*, and *Hnf4a*, to levels above or close to those present in eXEN cells (Fig. 1C; Supplemental Fig. S1C). Immunofluorescence analysis also confirmed that both *Gata4*-induced (data not shown) and *Gata6*-induced cells express Gata4, Gata6, Sox17, and Laminin proteins (Fig. 1D). Moreover, *Gata6*-induced cells no longer expressed Oct4 protein (Fig. 1D), suggesting that they have been reprogramed to iXEN cells.

In contrast, expression of *Sox7*, *Sox17*, *Hnf4a*, or *Foxa3* failed to induce a morphological switch to XEN-like cells within 6 d of induction (Fig. 1A). These factors inconsistently up-regulated ExEn genes and failed to up-regulate the expression of factors such as *Col4a1*, *Lama1*, or *Hnf4a* to eXEN cell levels (Fig. 1C; Supplemental Fig. S1C,D). We and others have previously observed that *Sox17*-overexpressing cells retained mES cell-like morphology and the expression of *Pou5f1*, *Sox2*, and *Nanog* after 48 h of induction (Niakan et al. 2010; McDonald et al. 2014). Similarly, *Sox17*, *Sox7*, or *Foxa3* levels of *Pou5f1*, *Nanog*, and *Sox2* were comparable with the expression in *dsRed* control cells after 6 d of induction (Fig. 1C). Moreover, *Sox7*-expressing colonies retain mES cell-like morphology and persistently express Oct4 protein despite a few Gata4-, Gata6-, Sox17-, and Laminin-expressing cells at the periphery of colonies (Fig. 1D).

We next induced transgene expression in the absence of LIF to determine whether destabilizing pluripotency via altered culture conditions would facilitate *Sox7*, *Sox17*, *Hnf4a*, or *Foxa3* induction of mES to iXEN cell reprograming within 6 d. Furthermore, as activation of FGF signaling is required for PrE development and derivation of XEN cells routinely involves addition of exogenous FGF (Feldman et al. 1995; Arman et al. 1998; Kunath et al. 2005; Chazaud et al. 2006; Yamanaka et al. 2010; Grabarek et al. 2012; Kang et al. 2013; Niakan et al. 2013), we also induced transgene expression in the presence of exogenous Fgf4 and heparin, which facilitates FGF receptor binding.

Although the induced cells lost their mES cell-like morphology, again, only *Gata6* or *Gata4* expression resulted in iXEN cell reprograming within the 6-d time period (Fig. 1A,C; Supplemental Fig. S1C–E). Additionally, we observed little difference in overall gene expression between the FGF-supplemented and LIF-deficient conditions, suggesting that exogenous FGF signaling does not enhance reprograming of mES cells to iXEN cells. In contrast *Sox7*-, *Sox17*-, *Hnf4a*-, or *Foxa3*-induced cells appeared to sporadically differentiate following LIF withdrawal. Despite the down-regulation of *Pou5f1*, *Sox2*, and *Nanog*, these cells did not exhibit XEN-like morphology and inconsistently up-regulated some but not all ExEn genes within this time frame. Altogether, this suggests that *Gata6* or *Gata4* induction is uniquely able to rapidly reprogram mES cells to iXEN cells. For subsequent experiments, we chose to use *Gata6* transgenic mES cells, as Gata6 mutant embryos exhibit an early PrE deficiency, and Gata6 is thought to lie upstream of Gata4 in the PrE transcriptional hierarchy (Morrisey et al. 1998; Koutsourakis et al. 1999; Chazaud et al. 2006; Plusa et al. 2008; Artus et al. 2011; Schrode et al. 2014). Furthermore, all inductions were performed in the presence of serum and LIF, as the absence of LIF alone destabilizes pluripotency gene expression.

### A short pulse of *Gata6* induction is sufficient to perturb the mES cell state

**Figure 2.**
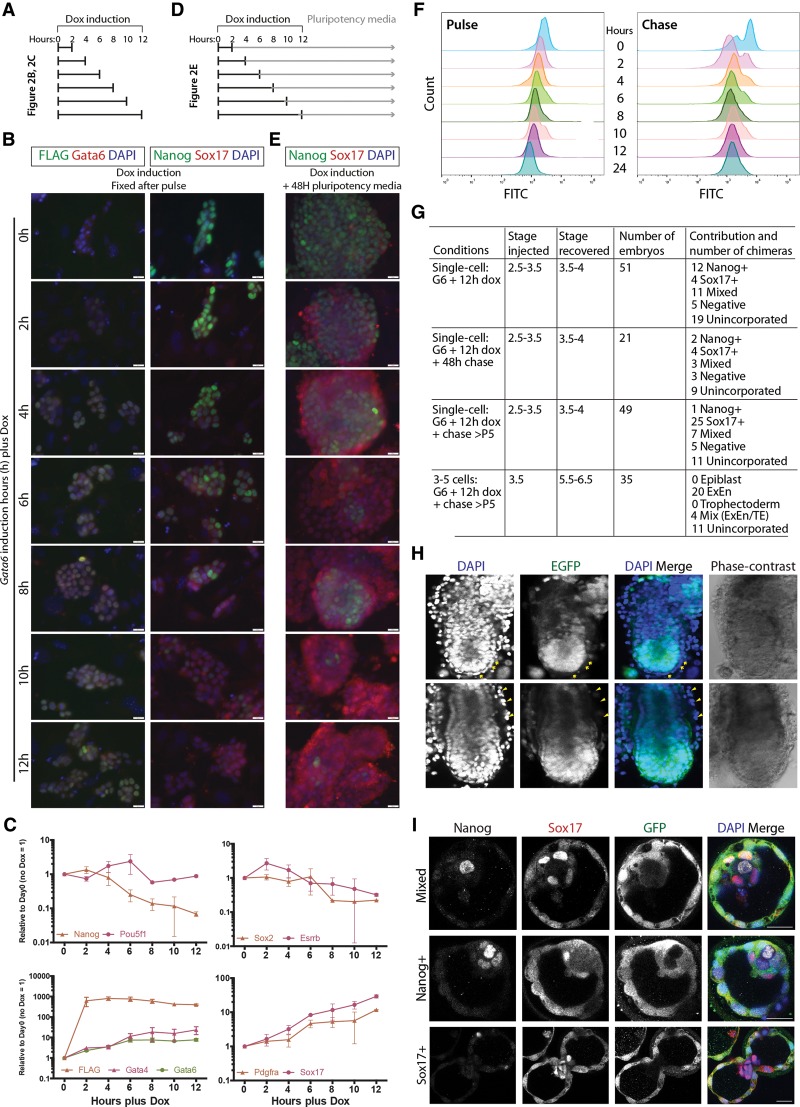
A short pulse of *Gata6* induction initiates iXEN cell reprograming (*A*) Time line of induction experiments. Cells were pulsed for incremental 2-h time periods and, at the end of each period, were analyzed by either immunohistochemistry, qRT–PCR, or flow cytometry. (*B*) Immunofluorescence analysis for Flag (green), Gata6 (red), and DAPI (blue) merge or with Nanog (green), Sox17 (red), and DAPI (blue) merge in *Gata6*-induced cells immediately after doxycycline treatment for the defined periods. Bars, 20 μm. (*C*) qRT–PCR analysis for exogenous Flag-tagged *Gata6* expression and selected pluripotency and endoderm transcripts in *Gata6*-overexpressing mES cells between 0 and 12 h of doxycycline induction. Relative expression is reflected as fold difference over uninduced mES cells normalized to *Gapdh*. Data are mean ± SEM of two biological replicates. (*D*) Time line of induction pulse-chase experiments. Cells were pulsed for incremental 2-h time periods and, at the end of each period, were switched into pluripotency medium for 48 h and then analyzed by immunohistochemistry or flow cytometry or switched into pluripotency medium to derive stable iXEN cells. (*E*) Immunofluorescence analysis for Nanog (green), Sox17 (red), and DAPI (blue) merge in *Gata6*-induced cells treated with doxycycline treatment for the defined periods and then switched into pluripotency medium for 48 h. Bars, 20 μm. (*F*) Flow cytometry analysis of Nanog expression at the defined time points in the pulse and pulse-chase cells. (*G*–*I*) Chimera contribution of unlabeled Gata6-induced cells injected into B5/EGFP embryos that constitutively express EGFP. (*G*) Summary of the number of cells injected, the stage of injection and dissection, and chimera contribution. (*H*) Embryonic day 5.5 (E5.5)–E6.5 post-implantation embryos with unlabeled iXEN cell contribution to the visceral (arrow) or parietal (arrowhead) endoderm. Representative images of chimeras with phase-contrast, DAPI (blue) nuclear staining, and embryo EGFP expression. (*I*) E3.5–E4 chimera blastocysts immunofluorescently analyzed for the expression of the PrE marker Sox17 (red), the epiblast marker Nanog (white), EGFP (green), and DAPI (blue) merge. Bars, 100 μm.

We next sought to investigate the minimum temporal requirement for Gata6 induction to affect mES cell gene expression. We induced exogenous expression for defined pulses of between 2 and 12 h and fixed the cells for immunohistochemistry immediately following doxycycline withdrawal (Fig. 2A). Flag and Gata6 protein expression was detectable by immunofluorescence and Western blot analysis 2–4 h following doxycycline addition (Fig. 2B; Supplemental Fig. S2A). Although induced cells retained mES cell morphology over the initial pulse period, we observed a clear effect on protein expression, with Nanog protein down-regulated 8–10 h following induction (Fig. 2B).

To examine gene expression dynamics in more detail, we performed qRT–PCR analysis between 2 and 12 h following *Gata6* induction (Fig. 2C). qRT –PCR amplification of the Flag region confirmed robust induction of exogenous Flag-tagged *Gata6* by 2 h after doxycycline induction and more than twofold down-regulation of *Nanog* by 6 h, followed by *Sox2* and *Esrrb* at 8 and 12 h, respectively. Western blot analysis over the 12-h period confirmed these dynamics, with steady down-regulation of Nanog (Supplemental Fig. S2A). However, *Pou5f1* expression was unchanged at these early time points (Fig. 2C). In contrast, endogenous *Gata6* and *Gata4* were up-regulated more than twofold relative to uninduced controls within 2 h of doxycycline induction. This was followed by greater than twofold up-regulation of *Sox17* and *Pdgfra* 4 and 6 h after induction, respectively.

We also followed the fate of pulse-induced cells for 48 h after they were returned to pluripotency maintenance medium in the absence of doxycycline (Fig. 2D). Over the 48-h period, cells induced from 6 to 12 h up-regulated Sox17 expression, suggesting that Gata6 had initiated the endogenous XEN program (Fig. 2E). Consistent with this, flow cytometry analysis of Nanog expression in Gata6 pulsed cells identified a gradual reduction of median Nanog expression with increased Gata6 induction time periods (Fig. 2F; Supplemental Fig. S3). This suggests that the induction of Gata6 leads to the inhibition of Nanog in most cells. Importantly, similar analysis in the pulse-chased cells reveals a similar shift and indicates that, by 8 h of exogenous Gata6 expression, most of the cells do not re-express Nanog (Fig. 2F; Supplemental Fig. S3). However, we occasionally observed some cells that retained Nanog expression (Fig. 2E). As heterogeneities in Nanog expression are known to exist within mES cell cultures (Chambers et al. 2007), this suggests that these cells likely have a higher threshold of pluripotency gene expression to overcome.

Stable iXEN cell lines were successfully derived from Gata6-expressing cells induced for 6–12 h and were maintained in the absence of doxycycline for >10 passages. qRT–PCR analysis confirmed that stable iXEN cells maintain XEN gene expression and do not re-express pluripotency genes (Supplemental Fig. S2B). We next sought to test the developmental potential of iXEN cells by generating chimera embryos. We injected unlabeled iXEN cells into B5/EGFP embryonic day 3.5 (E3.5) blastocysts that constitutively express EGFP (Hadjantonakis et al. 1998). We then analyzed chimera contribution in E5.5–E6.5 embryos (Fig. 2G,H). In the majority of chimera embryos (20 of 24), iXEN cells contributed to either the extraembryonic visceral or parietal endoderm, and we did not detect epiblast contribution. This confirms that, similar to previous studies using eXEN cells (Kunath et al. 2005), our iXEN cells were also capable of successful XEN contribution in chimera embryos.

To gain further insight into the developmental potential of iXEN cells, we introduced single unlabeled iXEN cells into B5/EGFP E2.5–E3.5 embryos via morula aggregation or blastocyst injection and analyzed chimera contribution at the blastocyst stage (Fig. 2G,I; Supplemental Fig. S2C). The majority of stable iXEN cells contributed to Sox17-expressing PrE cells in chimeras (25 of 38). Interestingly, when we introduced single Gata6 12-h pulse or 12-h pulse-chase cells, we found that while a similar number of chimera embryos had exclusive Sox17 expression, the 12-h pulse-chase cells had a lower contribution to Nanog-expressing cells (Fig. 2G,I; Supplemental Fig. S2C). This suggests that while Gata6 is potent and highly efficient in initiating iXEN cell reprograming, a sufficient time interval is required to commit to a XEN program.

**Figure 3.**
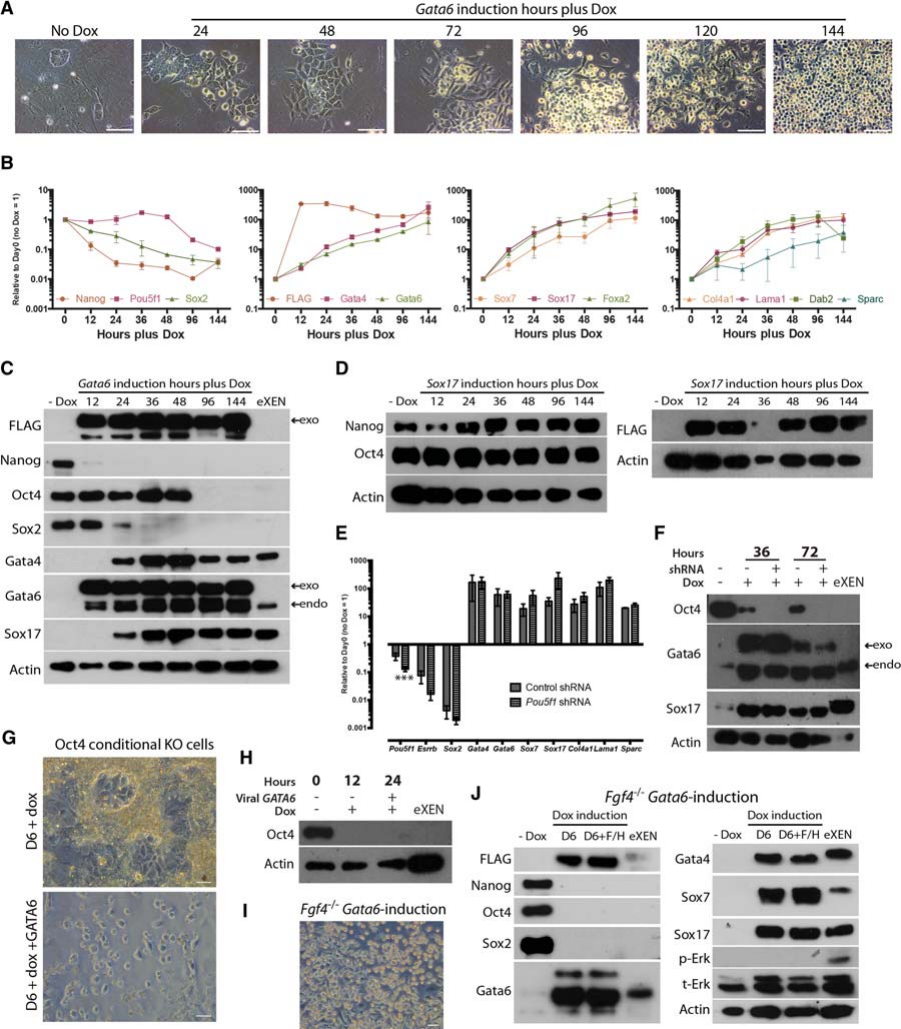
*Gata6* induction results in dynamic changes in cell morphology and gene expression even in the absence of Oct4 and Fgf4. (*A*) Representative phase-contrast images of *Gata6*-overexpressing mES cells at defined time points from 0 to 144 h of doxycycline treatment. Bars, 100 μm. (*B*) qRT –PCR analysis for selected pluripotency and endoderm transcripts in *Gata6*-overexpressing mES cells between 0 and 144 h of doxycycline induction. Relative expression is reflected as fold difference over uninduced mES cells normalized to *Gapdh*. Data are mean ± SEM of three biological replicates. (*C*) Western blot for selected proteins in *Gata6*-overexpressing cells from 0 to 144 h of doxycycline induction. A representative Actin loading control is included as a reference. Endogenous (endo) and exogenous (exo) Gata6 bands are indicated. (*D*) Western blot for selected proteins in *Sox17*-overexpressing cells from 0 to 144 h of doxycycline induction. A representative Actin loading control is included as a reference. (*E*) qRT –PCR analysis for selected pluripotency and endoderm transcripts following 72 h of shRNA knockdown of *Pou5f1* during *Gata6* induction compared with scrambled control shRNA. Data are mean ± SEM of five distinct shRNA constructs and two biological replicates. (***) *P* < 0.001. (*F*) Western blot for selected proteins in *Pou5f1* knockdown cells at the time points indicated. A representative Actin loading control is included as a reference. (*G*) Phase-contrast images of Oct4 conditional knockout cells following 6 d in the absence or presence of exogenous Gata6 induction. Bars, 20 μm. (*H*) Western blot for Oct4 protein in Oct4 conditional knockout cells at the time points indicated in the presence or absence of exogenous HA-tagged *GATA6*. A representative Actin loading control is included as a reference. (*I*) Phase-contrast image of stable *Fgf4*^−/−^ iXEN cells. Bars, 20 μm. (*J*) Western blot for selected proteins in *Fgf4*-null mES cells following 6 d of Gata6 induction in the absence or presence of Fgf4 (F) and heparin (H). A representative Actin loading control is included as a reference.

To further unravel the mechanisms of Gata6-mediated reprograming, we chose to investigate the effect of Gata6 induction over a 6-d (144-h) period, as we had previously observed complete down-regulation of Nanog, Oct4, and Sox2 by this time point. We analyzed defined time points between 12 and 144 h of induction to evaluate morphology and gene expression dynamics. From 24 h following *Gata6* induction, mES cell colonies changed from a domed to a flattened shape as cells migrated away from the center of the colony and eventually became refractile, rounded, and dispersed, similar to eXEN cells (Fig. 3A). qRT–PCR analysis confirmed that *Nanog* and *Sox2* transcripts were rapidly down-regulated within 12 h of doxycycline induction, with protein expression decreasing to levels below detection within 24 and 36 h, respectively (Fig. 3B,C). *Pou5f1* transcript and Oct4 protein displayed prolonged expression until 48 h after induction but were down-regulated by 96 h.

The qRT–PCR analysis also showed >10-fold up-regulation of ExEn genes between 12 and 48 h following doxycycline treatment (Fig. 3B). Interestingly, despite up-regulation of *Gata4* and *Sox17* transcripts within 12 h of *Gata6* induction (Fig. 2C), their proteins were not detectable by Western blot until 24 h following doxycycline treatment (Fig. 3C). Given that Nanog, Esrrb, and Sox2 are down-regulated in the absence of detectable Gata4 or Sox17 protein, this suggests that Gata6 directly mediates initial down-regulation of the pluripotency program. Despite robust induction of exogenous Sox17 over the 144-h period, these cells retained expression of Nanog and Oct4 (Fig. 3D) and show delayed up-regulation of endoderm factors compared with Gata6 (Fig. 1C). This is consistent with recent findings that Sox17-expressing cells only acquire XEN-like morphology and down-regulate pluripotency factor expression after 12 and 18 d of induction, respectively (McDonald et al. 2014). Altogether, this shows that Gata6 is uniquely able to rapidly down-regulate the core components of the pluripotency gene regulatory network and promote an ExEn program even in conditions that favor mES cell maintenance.

### Gata6 can induce reprograming of mES cells independently of Oct4 expression and Fgf4 signaling

Oct4 has been shown to be required for Fgf4-mediated PrE specification within the mouse embryo (Frum et al. 2013; Le Bin et al. 2014). Although Gata6 expression is initiated in Oct4 or Nanog mutant embryos, the subsequent absence of Sox17 and Gata4 suggests that PrE formation is compromised (Frankenberg et al. 2011; Frum et al. 2013; Le Bin et al. 2014). Given the persistent expression of *Pou5f1*/Oct4 following *Gata6* induction, we sought to investigate the interplay between Gata6, Oct4, and Fgf4 during mES cell reprograming.

To investigate the requirement for Oct4, we introduced shRNAs directed against *Pou5f1* simultaneously with doxycycline-induced expression of *Gata6* (Fig. 3E,F). We confirmed loss of *Pou5f1* expression by qRT –PCR (Fig. 3E) and Oct4 protein by Western blot analysis (Fig. 3F). Despite the premature loss of Oct4, *Gata6*-induced cells still up-regulated endogenous *Gata6*, *Sox17*, *Gata4*, *Lama1*, *Col4a1*, and *Sparc* and down-regulated *Sox2* and *Esrrb* (Fig. 3E). This suggests that Gata6 induction bypasses a possible requirement for Oct4 in reprograming mES cells to iXEN cells in vitro. To further confirm this, we used an Oct4 conditional knockout mES cell line that induces trophectoderm differentiation following doxycycline-dependent loss of Oct4 (Niwa et al. 2000; data not shown). We used a lentivirus to overexpress the human *GATA6* gene downstream from an *EF1*α promoter, which we confirmed successfully reprograms wild-type mES cells to iXEN cells (Supplemental Fig. S2D,E), similar to the doxycycline-inducible system. When we overexpressed *GATA6* in the Oct4-null cells, we observed the emergence of XEN-like cells (Fig. 3G), consistent with the *Pou5f1* knockdown cells. Western blot analysis confirmed that Oct4 protein was undetectable following 12 h of doxycycline treatment (Fig. 3H).

To determine whether *Gata6*-induced reprograming is dependent on the availability of endogenous Fgf4, we used site-specific recombination to integrate our inducible *Gata6* transgene into *Fgf4*^−/−^ mES cell lines (Cho et al. 2012; Kang et al. 2013). To eliminate the possibility of signaling from exogenous FGFs, we initially grew the *Fgf4*^−/−^ mES cells for three passages in serum-free medium in the presence of an Erk and Gsk3 inhibitor together with LIF (2i+LIF), as had been previously described (Ying et al. 2008). Interestingly, *Gata6* induction in *Fgf4*^−/−^ mES cells in basal serum-free medium in the absence of 2i+LIF or exogenous FGFs resulted in iXEN cell reprograming and the up-regulation of Gata4, Gata6, Sox7, and Sox17 (Fig. 3I,J), consistent with recent observations in mES cells transiently transfected with *Gata6* (Kang et al. 2013). Significantly, we also found that Oct4, Sox2, and Nanog were down-regulated independently of endogenous *Fgf4* following *Gata6* induction (Fig. 3J). To investigate the initial response to reprograming, we analyzed the *Fgf4*^−/−^ *Gata6*-induced cells at defined time points between 12 and 144 h of induction by qRT –PCR (Supplemental Fig. S2F). These cells exhibited similar expression dynamics for pluripotency gene down-regulation and ExEn gene up-regulation compared with *Fgf4*^+/+^ *Gata6*-induced cells (Fig. 3B) or *Fgf4*^−/−^ *Gata6*-induced cells treated with exogenous FGF and heparin (Supplemental Fig. S2F). *Fgf4*^−/−^ iXEN cell lines were maintained for >10 passages in the absence of exogenous *Gata6* without loss of gene expression (Supplemental Fig. S2G) or iXEN cell morphology (Fig. 3I), demonstrating that there appears to be no requirement for a feedback loop up-regulating Fgf4 to reinforce *Gata6*-mediated reprograming. Moreover, given the absence of phosphorylated Erk (Fig. 3J), it is unlikely that Erk signaling is required. In all, we found that neither Fgf4 nor Oct4 is required for *Gata6*-mediated reprograming of mES cells to iXEN cells.

### Gata6 directly regulates multiple components of the pluripotency gene regulatory network as well as ExEn genes

**Figure 4.**
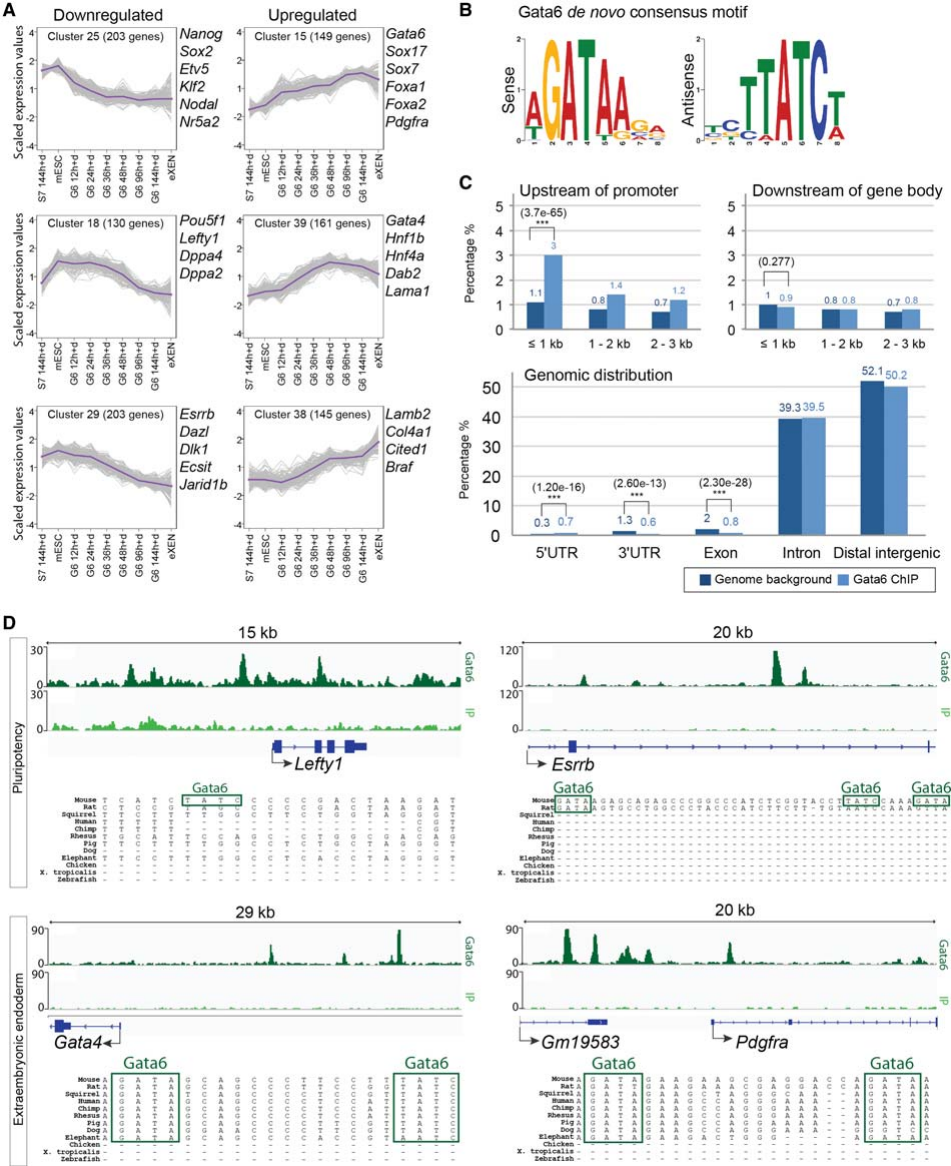
Gata6 enrichment near both pluripotency and endoderm genes. (*A*) Line plots of selected up-regulated or down-regulated clusters that contain key pluripotency or endoderm genes. Plots are based on mean scaled expression values from microarray analysis of *Gata6*-overexpressing cells from 12 to 144 h of doxycycline treatment. Uninduced mES cells, eXEN cells, and *Sox7*-overexpressing cells after 144 h of doxycycline treatment were also included. Genes were grouped into 50 clusters using K-means clustering according to normalized gene expression values scaled across time points. The trajectories of scaled expression values for individual genes in each cluster across time are shown as gray lines, while the purple lines correspond to the cluster mean. (*B*) MEME de novo motif analysis of the top Gata6-bound regions following ChIP-seq (ChIP followed by high-throughput sequencing) analysis. (*C*) Comparison of Gata6-binding distribution (light blue) at selected regions relative to the genomic average (dark blue). (***) *P* < 0.001. (*D*) ChIP-seq binding profiles showing Gata6 enrichment near pluripotency factors *Lefty1* and *Esrrb* and endoderm genes *Gata4* and *Pdgfra* (dark green). The input control profile (IP) is included for comparison (light green). Representative ChIP-seq binding profile of three biological replicates.

To characterize the global transcriptional profile during *Gata6*-mediated reprograming, we performed microarray analysis at defined time points from 12 to 144 h after induction. We included untreated mES cells to reflect the initial pluripotent state and eXEN cells as a reference for ExEn gene expression. Additionally, we included *Sox7*-expressing cells at 144 h after induction to compare their gene expression with *Gata6*-induced cells. To investigate gene expression dynamics, we performed K-means clustering on the scaled microarray data to group differentially expressed genes over the time course into 50 clusters (Fig. 4A; Supplemental Fig. S4).

We found that a number of functionally significant pluripotency-associated genes were rapidly and persistently down-regulated within 12 h of *Gata6* induction, including *Nanog*, *Sox2*, *Nr5a2*, *Klf2*, and *Nodal* (cluster 25) (Fig. 4A; Supplemental Fig. S4; Supplemental Table S1). Additional genes, including *Esrrb*, *Dazl*, *Dlk1*, *Ecsit*, and *Jarid1b* (cluster 29), were more gradually down-regulated over the time course compared with cluster 25. *Pou5f1*, *Lefty1*, *Dppa4*, and *Dppa2* (cluster 18) were more persistently expressed and eventually down-regulated, suggesting step-wise down-regulation of various nodes of the pluripotency gene regulatory network.

*Gata6* induction also up-regulated the expression of several ExEn transcription factors, cell surface proteins, and basement membrane components in a step-wise manner (Fig. 4A; Supplemental Fig. S4; Supplemental Table S1). Initial up-regulation of *Gata6*, *Sox17*, *Sox7*, *Foxa1*, *Foxa2*, and *Pdgfra* (cluster 15) was followed by up-regulation of *Gata4*, *Hnf1b*, *Hnf4a*, and the key cell surface and basement membrane components *Dab2* and *Lama1* (cluster 39), which are thought to confer an adherence difference to PrE cells (Gerbe et al. 2008; Niakan et al. 2010; Artus et al. 2011). Additional basement membrane components and ExEn genes, including *Lamb2*, *Col4a1*, *Cited1*, and *Braf* (cluster 38), were up-regulated later in the time course. We did not identify key markers of ectodermal (*Nestin*, *Pax6*, *Sox1*, and *Sox3*) or mesodermal (*Flk1*, *Hand1*, *Mixl1*, *Nkx2.5*, and *T*) lineages within our microarray data set, suggesting that Gata6 is specifically inducing an endoderm fate. In contrast, *Sox7*-expressing cells after 144 h of induction largely retained gene expression patterns similar to mES cells (Fig. 4A; Supplemental Fig. S4). Importantly, while *Sox7*-expressing cells have up-regulated some genes associated with XEN cell function, such as *Sall4* (cluster 33) (Lim et al. 2008), as before, they maintained expression of pluripotency factors (clusters 18, 25, and 29) and did not up-regulate endoderm-associated genes and basement membrane proteins to the same extent as *Gata6*-induced cells (clusters 15, 28, and 39).

Given the rapid changes in gene expression dynamics observed in our microarray analysis, we hypothesized that Gata6 may directly regulate both pluripotency and ExEn genes. We performed ChIP followed by high-throughput sequencing (ChIP-seq) analysis of Gata6 binding after 36 h of induction in order to capture both positive and negative gene regulatory dynamics as observed in our Western blot and qRT –PCR analyses (Fig. 3B,C). We identified 12,632 Gata6-bound regions enriched over the input control that were common between three biological replicates, with a false discovery rate (FDR) of <0.01% (Supplemental Table S2). We carried out de novo motif analysis on the top 500 most significant Gata6-bound regions and, as expected, identified the canonical GATA motif as the most highly enriched (Fig. 4B). We determined the binding distribution of Gata6 throughout the genome and found significant enrichment <1000 base pairs (bp) upstream of gene promoters compared with the whole genome (*P*-value ≤ 3.7 × 10^−65^). This is in contrast to Gata6 binding downstream from genes, which has no significant difference compared with the genome (*P*-value ≤ 0.277) (Fig. 4C).

Importantly, we found Gata6 binding enrichment at genes encoding multiple components of the pluripotency regulatory network, such as *Esrrb*, *Lefty1*, *Nr5a2*, *Nanog*, and *Pou5f1*, whose expression is down-regulated during reprograming (Fig. 4D; Supplemental Fig. S5; Supplemental Table S2). Gata6 was also enriched at a number of rapidly up-regulated ExEn-associated genes such as *Gata4* and *Pdgfra* (Fig. 4D; Supplemental Table S2), further suggesting that Gata6 directly regulates both pluripotency and ExEn genes. Intriguingly, the GATA motifs within these regions share a high degree of sequence conservation between placental mammals in some but not all cases. We also identified Gata6 enrichment upstream of *Fgfr2*, suggesting that Gata6 may be directly regulating FGF signaling (Supplemental Fig. S5; Supplemental Table S2).

To relate Gata6 binding to global gene expression dynamics, we compared the ChIP-seq analysis with our microarray cluster data set to identify the subset of Gata6-bound genes that were dynamically regulated over the microarray time course. We then performed gene ontology (GO) analysis comparing the Gata6-bound subset with the total microarray data set using the GOrilla analysis tool (Eden et al. 2009). Gata6-bound dynamically regulated genes were associated with signal transduction and regulation, DNA binding and transcriptional regulation, cell adhesion and migration, and stem cell maintenance (Supplemental Table S3). To investigate whether particular gene expression patterns correlated with GO, we identified a number of clusters that were continuously up-regulated or down-regulated over the Gata6 induction time course and compared their GO term enrichment patterns. Down-regulated genes (clusters 3, 6, 9, 18, 19, 20, 23, 24, 25, 29, 34, and 46) were associated with protein binding, sequence-specific DNA binding, and biosynthetic processes (Supplemental Table S3). In contrast, genes that were continuously up-regulated (clusters 11, 15, 30, 38, 39, 40, and 47) were enriched for membrane components, endoderm formation, and protein glycosylation (Supplemental Table S3).

Importantly, our ChIP-seq and time-course transcriptome analysis revealed genes whose expression is also rapidly down-regulated and that cluster with known pluripotency factors. Given that Gata6 directly regulates a number of known pluripotency factors, these additional genes may also function to maintain pluripotency. One such candidate is *Etv5* (cluster 25), whose expression has also been associated with mES cells (Zhou et al. 2007), but whose function has not yet been tested in this context. Consequently, our work may provide a useful resource to identify putative pluripotency or, conversely, endoderm factors. However, the absence of Gata6 enrichment near *Sox2*, whose expression is also rapidly down-regulated, may suggest indirect repression, possibly due to destabilizing alternative nodes of the pluripotency regulatory network. Alternatively, Gata6 may function as a repressor via a binding site located further away.

### Gata6 shares common gene targets and binding sites with pluripotency factors

**Figure 5.**
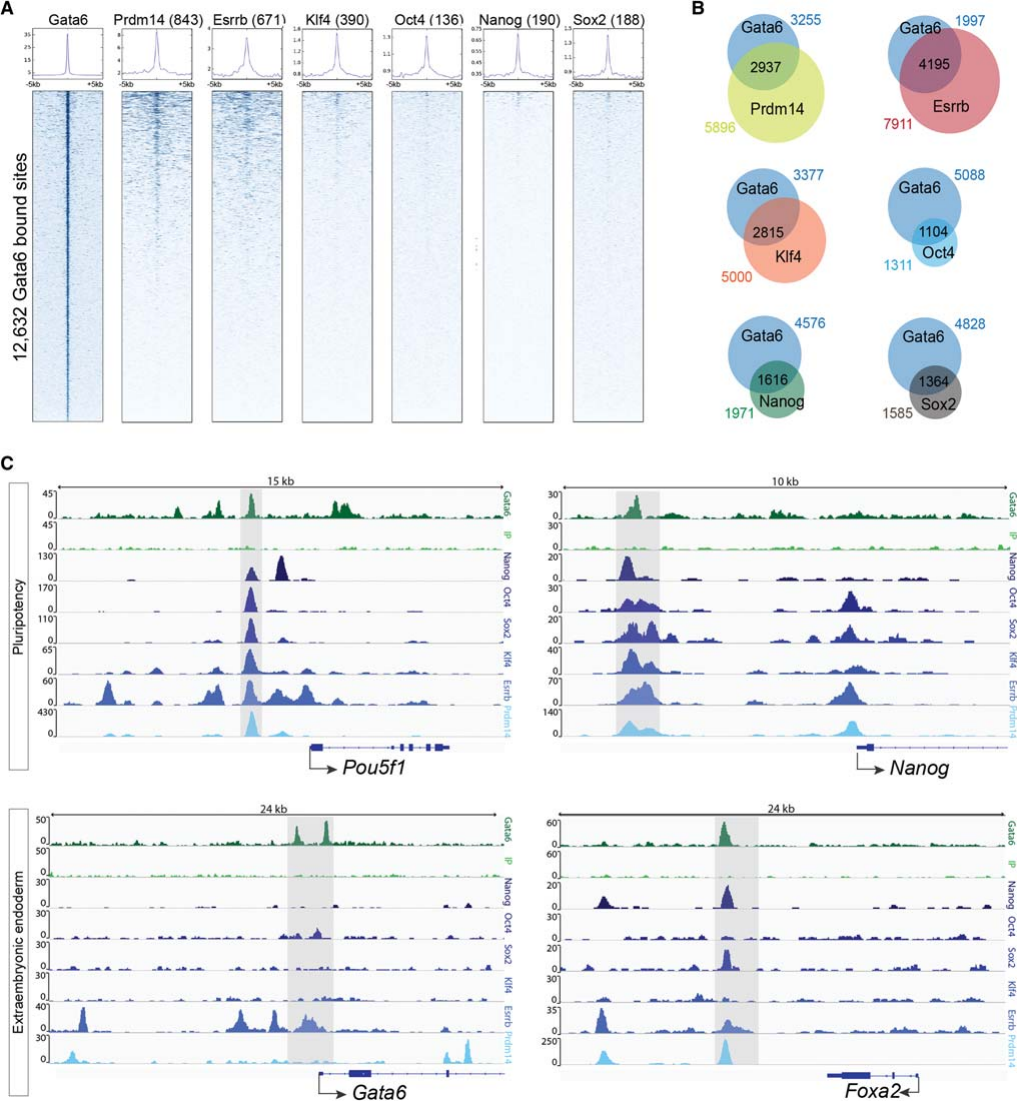
Gata6 binds to loci occupied by pluripotency factors in mES cells. (*A*) Density heat maps of Gata6-binding peak intensity after 36 h of Gata6 induction, indicating direct overlap with Nanog, Oct4, Sox2, Klf4, Esrrb, or Prdm14 binding in mES cells within a 10-kb window centered at the transcription start site (TSS). Data for Nanog, Oct4, Sox2, Klf4, Esrrb, and Prdm14 were obtained from the Mouse ES Cell ChIP-Seq Compendium (http://bioinformatics.cscr.cam.ac.uk/ES_Cell_ChIP-seq_compendium.html; Martello et al. 2012). (*B*) Venn diagram indicating the overlap of Gata6-bound genes during reprograming compared with genes previously shown to be bound by Oct4, Sox2, Klf4, Esrrb, or Prdm14 in mES cells. (*C*) Binding profiles at the *Pou5f1*, *Nanog*, *Gata6*, and *Foxa2* loci for Gata6 and the input control during reprograming compared with Nanog, Oct4, Sox2, Klf4, Esrrb, or Prdm14 in mES cells.

We next sought to determine whether Gata6 shares common gene targets and binding sites with pluripotency factors, which would suggest competition for pluripotency or ExEn target gene regulation. Using spatial heat map analysis, we compared Gata6-bound loci identified in our ChIP-seq analysis with published genome-wide occupancy of Oct4, Sox2, Nanog, Klf4, and Esrrb in mES cells (Fig. 5A; (http://bioinformatics.cscr.cam.ac.uk/ES_Cell_ChIP-seq_ compendium.html; Martello et al. 2012). We also investigated overlap between Gata6 and pluripotency factor gene targets (Fig. 5B).

We found that Gata6-bound loci directly overlapped with 136 Oct4, 190 Nanog, 390 Klf4, or 188 Sox2 gene loci (Fig. 5A). Intriguingly, some of these sites were present near pluripotency genes, including *Lefty1* (Supplemental Fig. S5), suggesting that Gata6 may directly compete with pluripotency factors to antagonize the regulation of some common gene targets. Interestingly, we identified common loci at the distal enhancers of *Pou5f1* and *Nanog* (Fig. 5C), which are known to be relevant for pluripotency regulation (Kagey et al. 2010). However, these sites form a small proportion of the total Gata6-bound loci. We found more extensive overlap between Gata6 and Esrrb (671 overlapping gene loci) (Fig. 5A), and shared loci were present in both pluripotency and ExEn genes (Fig. 5C). Notably, Gata6 and Esrrb were enriched at a common locus upstream of the endogenous *Gata6* promoter, suggesting possible competition for regulation.

We also observed that genes such as *Nr5a2* and *Esrrb* were bound by both Gata6 and one or more of the pluripotency factors but that these sites did not directly overlap (Supplemental Fig. S5). When we examined the shared gene target data set (Fig. 5B; Supplemental Table S4), we found that out of 6192 identified Gata6 target genes, 4195 were also targets of Esrrb, 2815 were also targets of Klf4, 1364 were also targets of Sox2, 1616 were also targets of Nanog, and 1104 were also targets of Oct4 (Fig. 5B), suggesting that Gata6 may regulate the pluripotency network both directly and indirectly. Curiously, we identified several key ExEn genes within the list of Gata6 gene targets shared with Esrrb, including *Gata6*, *Gata4*, *Sox17*, *Col4a1*, *Fgfr2*, *Pdgfra*, and *Sox7* (Supplemental Fig. S5; Supplemental Table S4). In contrast, most of these key ExEn genes were not identified among the Nanog, Oct4, and Sox2 target gene sets. Previous studies have shown that, in addition to its function in pluripotent cells downstream from Nanog (Festuccia et al. 2012), *Esrrb* knockdown or overexpression affects endoderm gene expression (Ivanova et al. 2006; Loh et al. 2006; Uranishi et al. 2013). However, when we overexpressed an *Esrrb* transgene (van den Berg et al. 2008; Uranishi et al. 2013) concomitant with doxycycline-induced *Gata6* expression, this did not prevent the down-regulation of Nanog expression (Supplemental Fig. S6A) or iXEN-like cell differentiation (data not shown). Moreover, by immunofluorescence analysis, we found that in mouse preimplantation embryos, Esrrb and Gata6 expression was coincident even after down-regulation of Nanog in Gata6-high PrE cells (Supplemental Fig. S6B). This suggests that Esrrb may not function to inhibit ExEn differentiation in this context or that alternative factors may be required in tandem to block the endoderm-promoting effect of Gata6. Knockdown of additional pluripotency factors such as *Prdm14* and *Nr5a2* has been shown to up-regulate ExEn genes (Ma et al. 2011; McDonald et al. 2014). Indeed, when we compared Gata6-bound loci with those occupied by Prdm14 in mES cells, we identified extensive overlap of common gene targets (Fig. 5B; Supplemental Table S4) as well as overlapping binding sites (843 overlapping gene loci) (Fig. 5A,C). Consequently, the greater degree of overlap with Gata6-bound sites may reflect the dual role of both Gata6 and a pluripotency factor subset in driving their respective cell fates.

To investigate whether the binding sites that we identified during Gata6 reprograming are maintained in eXEN cells, we performed ChIP-seq analysis of Gata6 occupancy in eXEN cell lines (Supplemental Table S5,S6). Of the 927 Gata6 gene targets in eXEN cells, 504 genes were also targets of Gata6 at the 36-h post-induction time point (Supplemental Table S4). This includes Gata6 endoderm target genes such as *Gata4*, *Gata6*, *Sox7*, and *Lamc1*. Interestingly, we did not detect enrichment of Gata6 binding near many of the pluripotency target genes that we identified at the 36-h post-induction time point, with the notable exception of *Nr5a2*. This demonstrates that Gata6 binding in eXEN cells is distinct compared with cells in transition and suggests that there may not be a requirement for Gata6 to actively repress pluripotency factor expression long after they are down-regulated.

### Gata6 initiates an ExEn program in differentiated cells and hES cells

**Figure 6.**
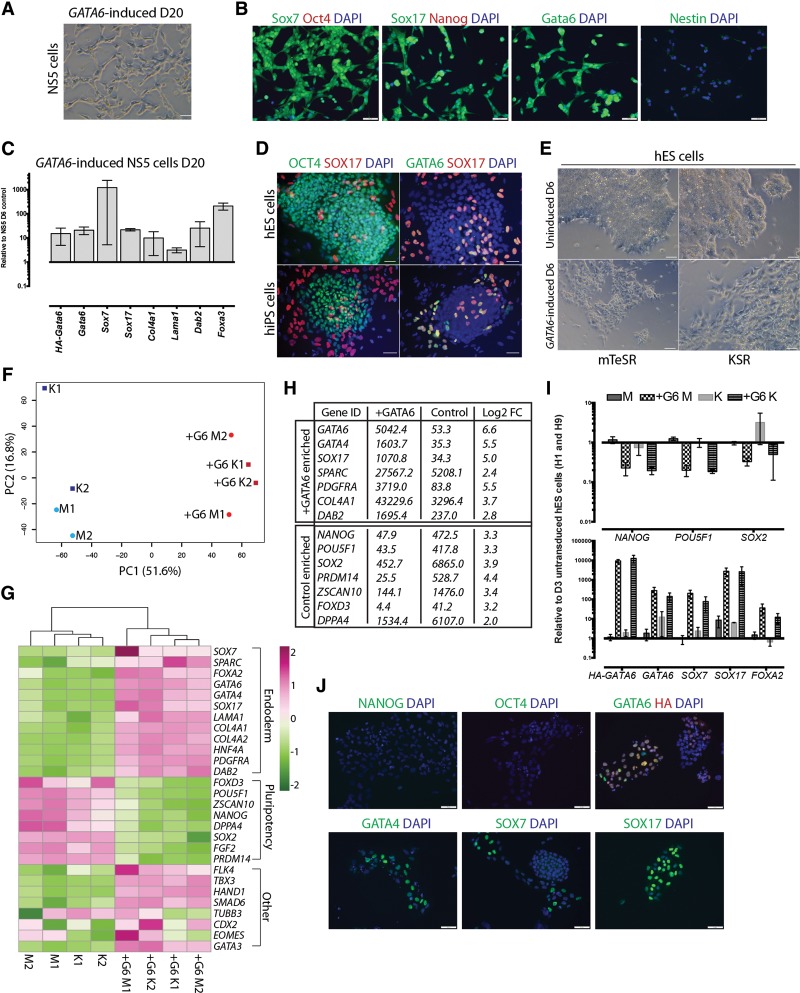
Gata6 initiates an ExEn program in mouse neural stem cells and hES cells. (*A*) Phase-contrast images of uninduced or *GATA6*-induced mouse neural stem cells. Bars, 50 μm. (*B*) Immunofluorescence analysis for Sox7, Sox17, Gata6, or Nestin (green) and Oct4 or Nanog (red) with DAPI (blue) merge in *GATA6*-induced mouse neural stem cells after 20 d of induction. Bars, 50 μm. (*C*) qRT –PCR analysis of mouse neural stem cells for HA-tagged exogenous *GATA6* and selected endoderm transcripts (*Gata6*, *Sox7*, *Sox17*, *Col4a1*, *Lama1*, *Dab2*, and *Foxa3*) 20 d following transduction. Data are mean ± SEM of two replicates. (*D*) Immunofluorescence analysis for OCT4 or GATA6 (green) and SOX17 (red) with DAPI (blue) merge in hES and human iPS (hiPS) cells in pluripotent culture conditions. Bars, 50 μm. (*E*) Representative phase-contrast images of *GATA6*-transduced hES cells 6 d after *GATA6* induction compared with uninduced controls in pluripotency (mTeSR) or differentiation (KSR) medium. Bars, 50 μm. (*F*) Principal component analysis using RPKM (reads per kilobase per million mapped reads)-normalized RNA sequencing (RNA-seq) data from biological replicates of uninduced and *GATA6*-induced (+G) cells in KSR (K) or mTeSR (M) medium. (*G*) Heat maps showing the hierarchical clustering of uninduced and *GATA6*-induced (+G) cells in KSR (K) or mTeSR (M) medium using RPKM-normalized RNA-seq data. Expression levels plotted on a high-to-low scale (purple–white–green). (*H*) DESeq analysis indicating genes significantly differentially expressed in the uninduced control versus GATA6-induced cells in both the KSR and mTeSR conditions. The median gene expression and log_2_ fold change (FC) difference in expression are noted. (*I*) qRT –PCR analysis of H1 and H9 hES cells for HA-tagged exogenous *GATA6* expression and selected pluripotency (*POU5F1*, *NANOG*, and *SOX2*) and endoderm (*GATA6*, *SOX7*, *SOX17*, and *FOXA2*) transcripts 6 d following transduction. Data are mean ± SEM of two to three replicates. (*J*) Immunofluorescence analysis of OCT4, NANOG, GATA4, SOX17, SOX17, and GATA6 (all green) and HA (red) with DAPI (blue) merge in *GATA6*-induced H9 hES cells 5 d following doxycycline treatment. Bars, 50 μm.

We also sought to determine whether Gata6 could reprogram cells other than mES cells. To investigate this, we used a pure culture of stable neural stem cells that were previously shown to generate functional neurons that contribute to the adult brain in mouse chimeras, without the formation of teratomas (Conti et al. 2005). This avoids the possibility of residual pluripotent or partially differentiated cells, which may be present at early stages of directed differentiation protocols. We expressed *GATA6* in neural stem cells by lentiviral transduction in neural basal medium and evaluated the identity of the cells 20 d after induction (Fig. 6A). Despite their morphological resemblance to neurons, immunofluorescence analysis confirms robust induction of Gata6, Sox7, and Sox17 proteins (Fig. 6B) in the GATA6-overexpressing cells. Importantly, the preinduced cells lacked detectable expression of Oct4 and Nanog (Conti et al. 2005), which we confirmed was also undetectable in the GATA6-induced cells (Fig. 6B). We observed low levels of detectable Nestin expression in these cells (Fig. 6B), suggesting that an additional time interval may be required to stabilize the XEN cell program and fully overcome the neural stem cell state. qRT –PCR analysis confirmed that the *GATA6*-induced cells up-regulated the XEN factors *Gata6*, *Sox7*, *Sox17*, *Col4a1*, *Lama1*, *Dab2*, and *Foxa3* (Fig. 6C). In all, the ability of Gata6 to promote reprograming to iXEN-like cells does not appear to be restricted solely to mES cells.

We next sought to investigate the broader reprograming potential of Gata6. We observed SOX17 and GATA6-coexpressing cells within hES and iPS cell cultures maintained in pluripotency conditions (Fig. 6D). This is reminiscent of the Sox17-expressing XEN-committed cells that we observed previously in mES cell cultures (Niakan et al. 2010) and suggests that hES cell may also have the potential to be converted to XEN cell lines. We then transduced hES cells in both pluripotency (mTeSR) and differentiation-promoting (KSR) conditions to determine whether *GATA6* expression is sufficient to drive iXEN cell reprograming from hES cells. Significantly, as stable human XEN cell lines have yet to be established, no morphological benchmark exists.

*GATA6*-transduced hES cells exhibited a morphology that is distinct from hES cell colonies even in conditions that otherwise favor their pluripotency (Fig. 6E). We next compared the global transcriptional profile of *GATA6*-transduced versus untransduced hES cells by RNA sequencing (RNA-seq). We initially used principal component analysis (PCA), which demonstrates that the *GATA6*-induced samples cluster together irrespective of the basal medium and were transcriptionally distinct from untransduced cells (Fig. 6F). We also used an independent method of hierarchical clustering, which again demonstrates that, independent of the basal medium, the *GATA6*-induced cells cluster together and are distinct from the untransduced cells (Fig. 6G). Notably, *GATA6*-transduced hES cells up-regulated a number of extraembryonic and/or pan-endoderm factors, including *GATA6*, *GATA4*, *SOX17*, *SOX7*, *FOXA2*, *PDGFRA*, *COL4A1*, *COL4A2*, *LAMA1*, *HNF4a*, *DAB2*, and *SPARC* (Fig. 6G,H; Supplemental Tables S5, S6). Significantly, we confirmed that the *GATA6*-induced cells down-regulated pluripotency factors, including *NANOG*, *POU5F1*, *SOX2*, *PRDM14*, *FGF2*, *FOXD3*, *DPPA4*, and *ZSCAN10*. qRT–PCR analysis confirmed the up-regulation of endogenous *GATA6*, *SOX17*, *SOX7*, and *FOXA2* transcripts between 10-fold and 1000-fold and the down-regulation of the expression of *NANOG*, *SOX2*, and *POU5F1* (Fig. 6I). However, expression of genes associated with alternative lineages, including *FLK4*, *TBX3*, *CDX2*, *GATA3*, *SMAD6*, *EOMES*, *HAND1*, and *TUBB3*, suggests that the *GATA6*-induced hES cells may not be fully reprogramed to stable iXEN cells (Fig. 6I). Moreover, although we clonally passaged the *GATA6*-transduced hES cells more than three times and they maintained their morphology for >1 mo, they could not be maintained indefinitely, suggesting that alternative conditions or factors may be required to derive stable human iXEN cells.

In tandem, we engineered GATA6 doxycycline-inducible hES cells by lentiviral transduction of a tetracycline/doxycycline-inducible HA-tagged *GATA6* transgene. hES cells in pluripotency medium treated with doxycycline exhibited a morphological switch similar to the *GATA6* virally transduced cells (Fig. 6E; data not shown). Importantly, the *GATA6* doxycycline-induced cells have down-regulated OCT4 and NANOG in most cells (Fig. 6J). Moreover, the induced cells have up-regulated the expression of the HA-tagged GATA6, SOX7, SOX17, and GATA4 proteins (Fig. 6J). While exogenous GATA6 initiated reprograming of hES cells to iXEN-like cells, the heterogeneity in endoderm protein induction suggests that an additional time interval, culture condition, or factor may be needed to generate self-renewing human iXEN cell lines. Nevertheless, this suggests that GATA6/Gata6 is sufficient to overcome a number of distinct cell states to drive iXEN-like cell reprograming.

## Discussion

We show that Gata6 functions to reprogram a number of cell types into iXEN-like cells. In the context of mES cell reprograming, it does so by rapid and potentially direct repression of the pluripotency gene regulatory network coupled with activation of an endoderm gene program. What distinguishes Gata6 from other endoderm transcription factors that we tested is the speed with which it acts to induce a cell fate switch in the absence of selection. Indeed, most reprograming, including iPS cells, can take several days of selective culture. As the induction of Sox17 takes >2 wk to down-regulate pluripotency (McDonald et al. 2014), this suggests that indirect mechanisms eventually lead to XEN conversion, perhaps via Gata6. It would therefore be interesting to determine whether Sox17 can induce XEN reprograming in the absence of Gata6. This seems unlikely given that Gata6 mutant mES cells fail to initiate cXEN cell conversion in growth factor-mediated conditions, in contrast to Sox17 mutant ES cells that initiate but fail to maintain cXEN cells (Cho et al. 2012).

**Figure 7.**
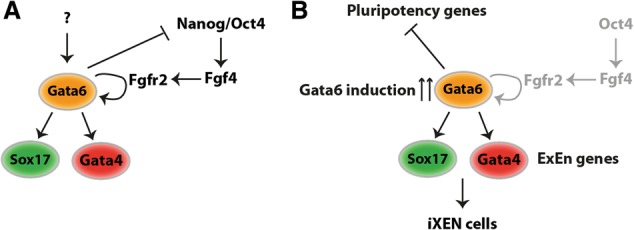
Model of the hierarchy of gene activity during iXEN reprograming. Here we show that the requirement for Oct4/Nanog during development to reinforce PrE development via FGF signaling (*A*) can be bypassed in vitro with the induction of Gata6 (*B*), which can potently induce mES-to-iXEN cell reprograming in the absence of Oct4 or Fgf4. Gata6 can bind to and rapidly induce the expression of downstream elements of an ExEn gene regulatory network (including Gata4 and Sox17). Gata6 can also simultaneously inhibit the expression of core and peripheral components of the pluripotency gene regulatory network. In all, the dual function of Gata6 as a repressor and activator potently drives mES-to-iXEN cell reprograming.

Investigating the mechanisms of *Gata6*-mediated iXEN cell reprograming may provide insights into the genetic hierarchy involved in ExEn development in vivo (Fig. 7). In mouse embryos, Gata6 expression is initiated in the absence of Oct4/Nanog-mediated Fgf4 signaling, but downstream Gata4 and Sox17 expression is compromised (Frankenberg et al. 2011; Frum et al. 2013; Le Bin et al. 2014; Schrode et al. 2014). Our results suggest that induction of Gata6 can up-regulate Gata4 and Sox17 in the absence of Fgf4 in vitro, consistent with recent findings (Kang et al. 2013). Ectopic expression of Gata6 has also been shown to restore endoderm differentiation in ES cells lacking the FGF signaling adaptor Grb2 (Wang et al. 2011). We also show that Gata6 positively regulates itself as well as Fgfr2, Gata4, and Sox17. This suggests that, in the embryo, Gata6 may require a feedback loop via Fgf4/Fgfr2 signaling to reinforce its own expression to achieve a certain threshold, which subsequently triggers the expression of Gata4 and Sox17. This is consistent with the insufficiency of exogenous Fgf4 to restore Sox17 expression in Gata6 mutant embryos (Schrode et al. 2014). This may also explain the colocalization of Nanog and Gata6 in vivo, whereby a threshold of Gata6 expression needs to be reached in order to overcome pluripotency and specify the PrE. Gata6 overexpression in vitro likely exceeds this threshold, thereby leading to down-regulation of pluripotency and up-regulation of ExEn genes, thus allowing iXEN reprograming to proceed in the absence of Fgf4. Inducing high levels of Gata6 expression in Fgf4 mutant mouse embryos would be one approach to test this hypothesis. Alternatively, Gata6 levels could be fine-tuned in vitro to determine whether Gata6-low *Fgf4* mutant cells fail to induce Gata4 and Sox17. Lowering the dose of doxycycline in our inducible system has been shown to reduce the penetrance and therefore the percentage of cells inducing expression rather than the quantitative levels within a given cell (Beard et al. 2006). This apparent discrepancy between the in vivo requirement for Fgf4 signaling in the PrE may also be the result of the presence of signaling pathways that facilitate the destabilization of pluripotency in vitro or differences between ES cells and early inner cell mass cells within the blastocyst (Boroviak et al. 2014).

One of the advantages of our analysis of global Gata6 binding during the transition of mES cells to iXEN cells is that it revealed that Gata6 may function simultaneously as both an activator and repressor of genes during reprograming but not in eXEN cells. It has been suggested that other GATA transcription factors, such as Gata1, also have a dual activator and repressor role (Yu et al. 2009). However, it is unclear precisely how Gata6 functions to regulate both sets of target genes and whether cofactors, chromatin, or other epigenetic mechanisms may influence this decision. The analysis of sequences surrounding Gata6-bound loci has not yet revealed motifs that consistently distinguish Gata6-bound down-regulated genes from Gata6-bound up-regulated genes (data not shown). Interestingly, we identified Gata6 binding upstream of the *Nanog* promoter and within an intron of *Esrrb*. One possibility is to use the emerging RNA-guided CRISPR–Cas nuclease system (Cong et al. 2013; Mali et al. 2013) to mutagenize these endogenous sites to interrogate not only these putative Gata6 regulatory loci but also those of other pluripotency factors and endoderm targets.

Remarkably, Gata6 drives iXEN-like cells from mouse neural stem cells, showing that it is a broad inducer of reprograming. It is surprising that Gata6 can overcome intrinsic programs within these cell types. As mES cells have a characteristic open chromatin state, Gata6 may have fewer roadblocks to directly bind to regulatory elements to control gene expression. However, in neural cells, it would be surprising if all endoderm target genes remained readily accessible for Gata6 direct regulation. Therefore, one possibility is that Gata6 may be functioning as a pioneering transcription factor in these contexts, exposing otherwise closed heterochromatic regions, as has been shown for FoxA2 and Gata4 (Zaret and Carroll 2011). Given the pleiotropic function of Gata6 in promoting endoderm- and mesoderm-derived cell types, it is also surprising that there is only one reprograming outcome. Further characterization of Gata6-mediated reprograming in several cellular contexts would allow interrogation of the relationship between transcription factors, signaling, and epigenetics in driving cell state transitions.

Induction of *SOX7* or *SOX17* has been previously reported to drive XEN-like or definitive endoderm-like cells, respectively, from hES cells (Seguin et al. 2008). However, these cells cannot be maintained indefinitely in culture, and, significantly, *SOX7*- or *SOX17*-expressing hES cells retain pluripotency gene expression (Seguin et al. 2008). GATA6 induction is able to both inhibit the pluripotency program and promote ExEn gene expression, suggesting that, in this context, stable human XEN cells may have the potential to be established. Given the potency of the doxycycline-inducible system in initiating iXEN reprogramming in hES cells, it would be interesting to determine whether alternative culture conditions could effectively capture stable human iXEN cells. Recent transcriptomic analysis of human embryos (Yan et al. 2013) may lead to the identification of signaling pathways that may be important to stabilize GATA6-induced human XEN cell lines. Together, this demonstrates that Gata6 is a versatile and potent reprograming factor that can act alone to drive a cell fate switch from diverse cell types.

## Materials and methods

### Culture conditions for pluripotent stem cell lines and transcription factor induction

mES cells were maintained on mouse embryonic fibroblast (MEF)-coated pregelatinized tissue culture plates (Corning) in serum and 10 ng/mL LIF. Additional medium components are listed in Supplemental Table S4. For details of the generation of inducible mES cell lines, see the Supplemental Material. Induction of mES cells was performed in pluripotency maintenance medium using doxycycline at a final concentration of 1 µg/mL. Doxycycline (1 µg/mL) was also used to generate Oct4-null cells from ZHBTc4 mES cells (Niwa et al. 2000). H9 and H1 hES cells (WiCell) were cultured in mTeSR1 (Stem Cell Technologies) on Matrigel (BD Biosciences)-coated dishes. Lentiviral packaging was performed in HEK293T cells using Lipofectamine 3000 (Life Technologies) cotransfection of a plasmid containing an *EF1*α promoter driving the expression of human HA-tagged GATA6 and the puromycin resistance gene (AMSbio) together with packaging plasmids. Forty-eight hours after lentiviral transduction, cells were selected using 1 µg/mL puromycin (Sigma).

### qRT–PCR

RNA was isolated using TRI reagent (Sigma) and DNase I-treated (Ambion). cDNA was synthesized using a Maxima first strand *cDNA* synthesis kit (Fermentas). qRT –PCR was performed using Quantace Sensimix on an Applied Biosystems 7500 machine (Life Technologies Corporation). Primer pairs were previously published (Molkentin et al. 1997; Fujikura et al. 2002; Niwa et al. 2005; Brown et al. 2010a) or designed using Primer3 software. All primers are listed in Supplemental Table S6.

### Immunohistochemistry and imaging

Samples were fixed in 4% paraformaldehyde for 1 h or overnight at 4°C, permeabilized with 0.5% Tween in 1× PBS for 20 min, and blocked with 10% FBS diluted in 0.1% Tween in 1× PBS for 1 h. Primary antibodies were diluted at 1:500 in blocking solution, and samples were incubated overnight at 4°C rotating. Secondary antibodies were diluted at 1:300 in blocking solution, and samples were incubated for 1 h at room temperature, washed, and covered with 0.1% Tween in 1× PBS containing DAPI VectaShield mounting medium (Vector Laboratories). A list of the antibodies used is in Supplemental Table S7. Images were taken on either an Olympus 1X71 microscope with Cell^F software (Olympus Corporation), a Zeiss Axiovert 200M microscope with AxioVision release 4.7 software (Carl Zeiss Ltd.), or a Leica SP5 inverted confocal microscope (Leica Microsystems Ltd).

### Western blot analysis

Whole-cell protein was extracted with CelLytic M reagent (Sigma) supplemented with proteinase and phosphatase inhibitors (Roche). Thirty micrograms of protein per sample was resolved on 12% SDS-PAGE gels and transferred to PVDF membrane using a Bio-Rad Trans-Blot transfer system (Bio-Rad). Membranes were blocked in 5% skimmed milk or 5% BSA in TBS 0.1% Tween and incubated with primary antibody overnight at 4°C. Following washes in TBS 0.1% Tween, membranes were incubated with secondary antibody in 5% milk or 5% BSA for 1 h at room temperature. Proteins were visualized using the Pierce ECL Western blotting substrate (Thermo). Antibodies used are listed in Supplemental Table S7.

### Microarray analysis

Total RNA was isolated as above and DNase I-treated (Ambion). RNA quality was assessed on a eukaryote total RNA Nano series II (Agilent Technologies) and then processed on an Agilent 2100 Bioanalyzer using the RNA electrophoresis program. All RNA samples were amplified using the Total Prep 96 RNA amplification kit (Ambion). Samples were hybridized to Illumina MouseWG-6_V2 expression BeadChip arrays (Illumina, Inc.) Biological triplicates were collected for each sample. Computational analysis details are included in the Supplemental Material.

### ChIP-seq

Gata6-inducible mES cells were seeded at 1 × 10^4^ cells per square centimeter and treated with 1 µg/mL doxycycline for 36 h prior to harvesting. Immunoprecipitation was performed on 1 × 10^7^ to 2 × 10^7^ cells as described (Vokes et al. 2007) for three biological replicates versus input samples. Sonication was performed using a Misonix 4000 (28 cycles of 15 sec on and 45 sec off at an intensity of 70%) with a microtip probe (Misonix). The antibodies used are listed in Supplemental Table S7. Libraries were prepared using the TruSeq ChIP sample preparation kit, and the resulting samples were sequenced using the Illumina Genome Analyzer II (Illumina). Data will be deposited into Gene Expression Omnibus and released immediately after publication (GSE69323). Computational analysis details are included in the Supplemental Material.

## Supplementary Material

Supplemental Material
