# Supplementary material for: Gata6 potently initiates reprograming of pluripotent and differentiated cells to extraembryonic endoderm stem cells
Source: Genes Dev. 2015 Jun 15;29(12):1239–55. doi: 10.1101/gad.257071.114 (PMC4495396; doi:10.1101/gad.257071.114)
Supplement: Supplemental Material [file supp_29_12_1239__index.html]

Supplemental Material 

# Gata6 potently initiates reprograming of pluripotent and differentiated cells to extraembryonic endoderm stem cells

## Supplemental Material

**Files in this Data Supplement:**

- Supp Fig S1.tif
- Supp Fig S2.tif
- Supp Fig S3.tif
- Supp Fig S4.tif
- Supp Fig S5.tif
- Supp Fig S6.tif
- Supp Material.docx
- Supp Table S1.xls
- Supp Table S2.xls
- Supp Table S3.xlsx
- Supp Table S4.xlsx
- Supp Table S5.xlsx
- Supp Table S6.xlsx
- Supp Table S7.xlsx
- Supp Table S8.xlsx
