# Supplementary figures and images for: Gata6 potently initiates reprograming of pluripotent and differentiated cells to extraembryonic endoderm stem cells
Source: Genes Dev. 2015 Jun 15;29(12):1239–55. doi: 10.1101/gad.257071.114 (PMC4495396; doi:10.1101/gad.257071.114)

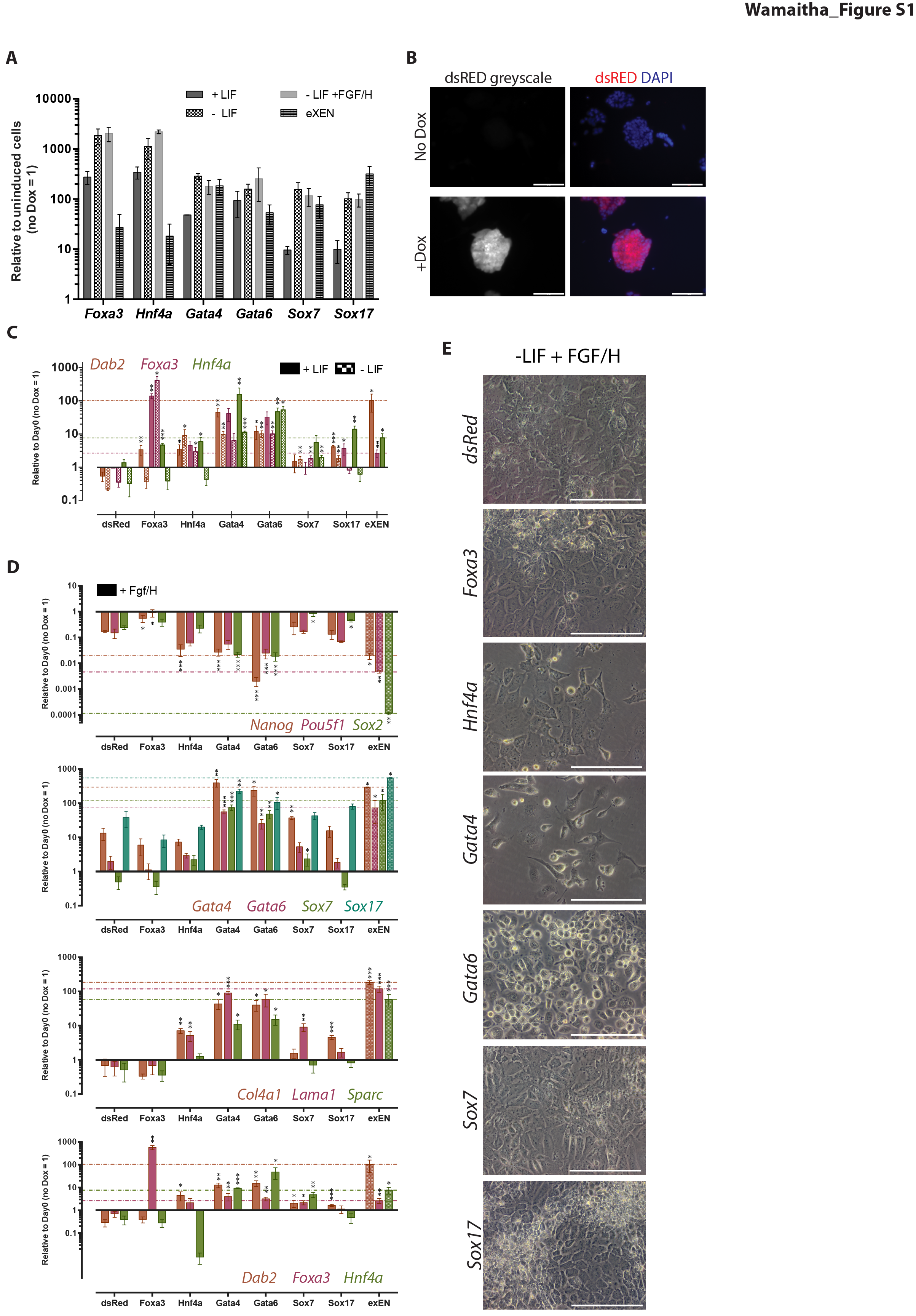

Supplement: Supplemental Material [file supp_29.12.1239_Supplemental_Figure_S1.tif]

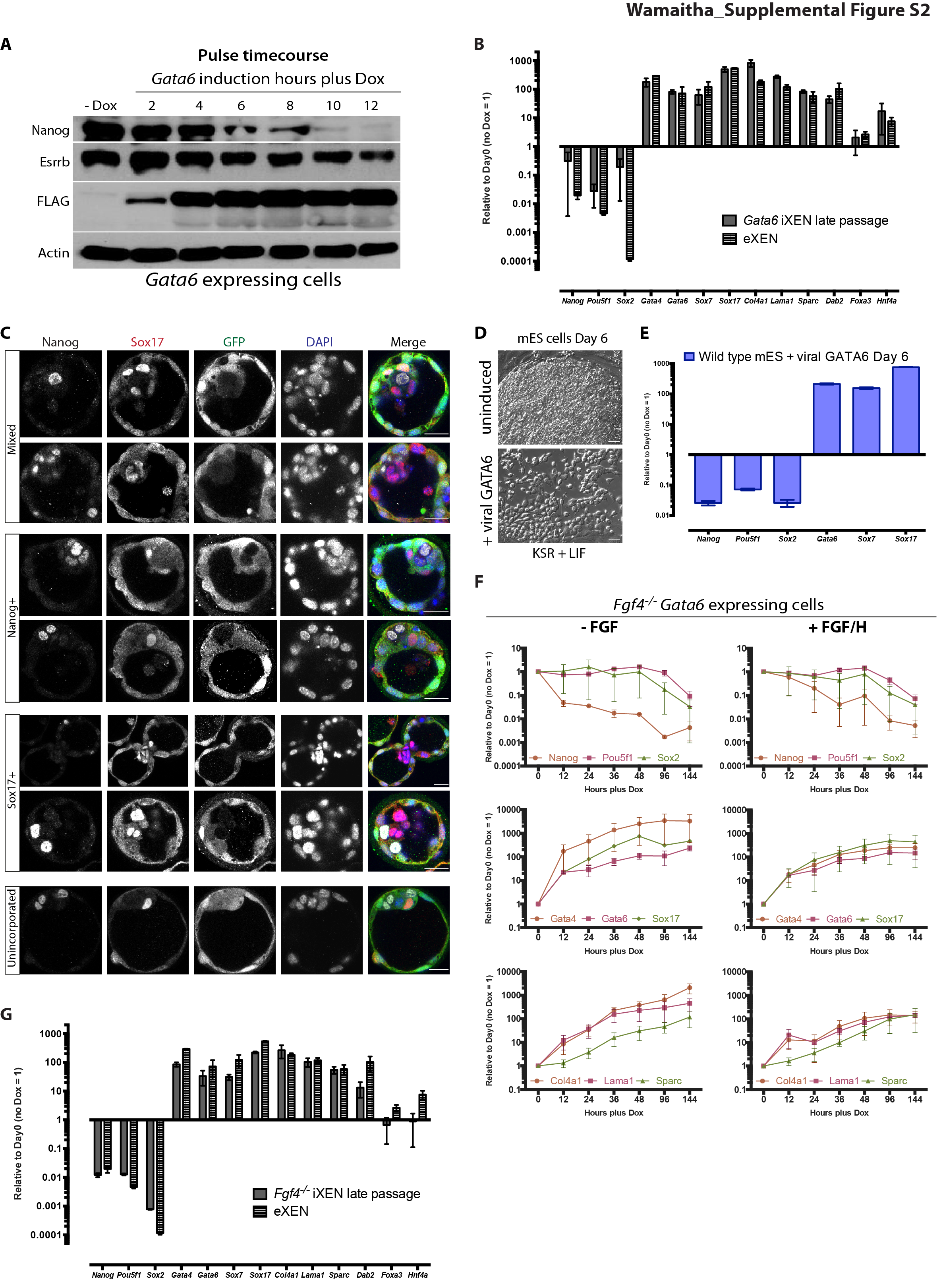

Supplement: Supplemental Material [file supp_29.12.1239_Supplemental_Figure_S2.tif]

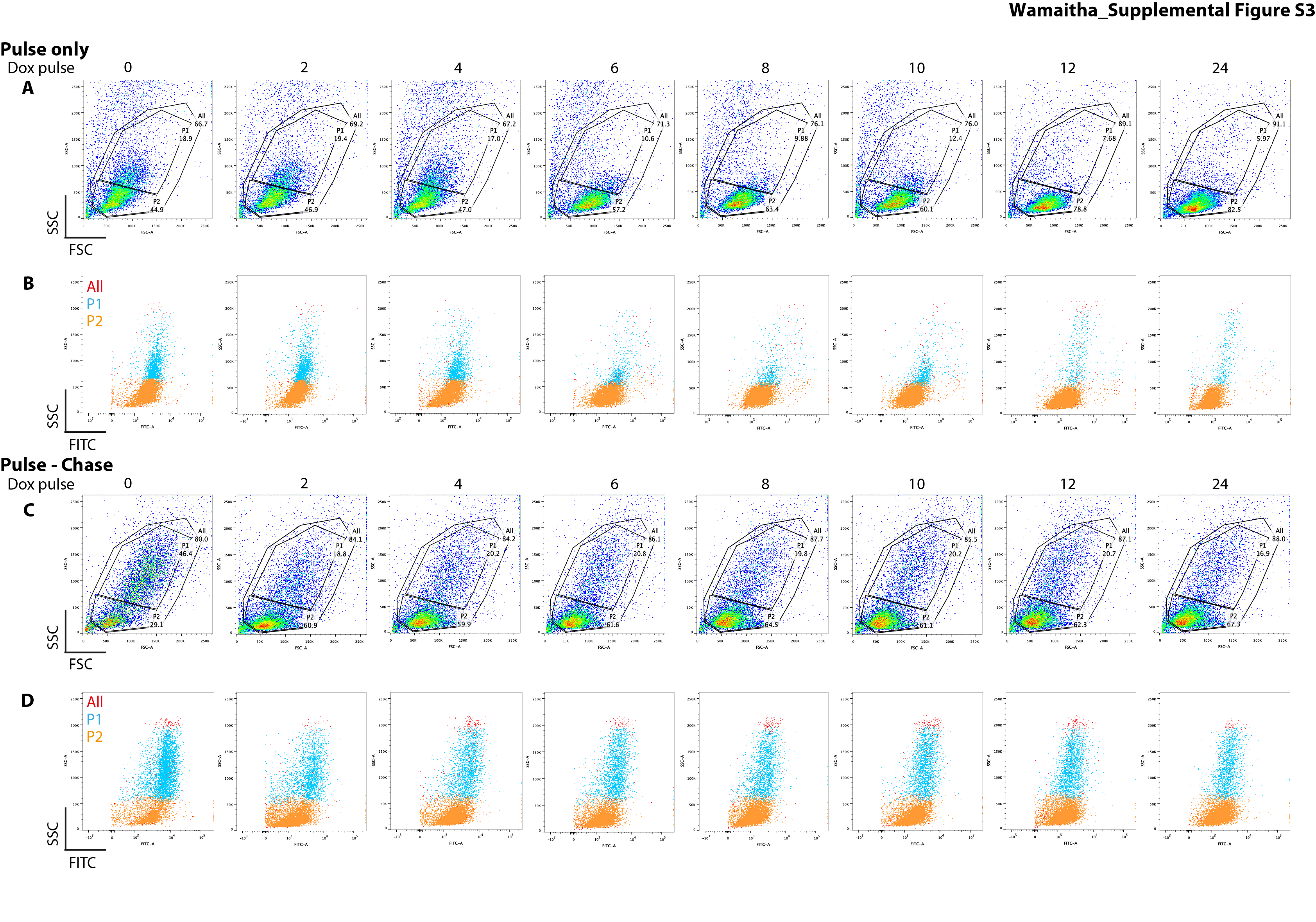

Supplement: Supplemental Material [file supp_29.12.1239_Supplemental_Figure_S3.tif]

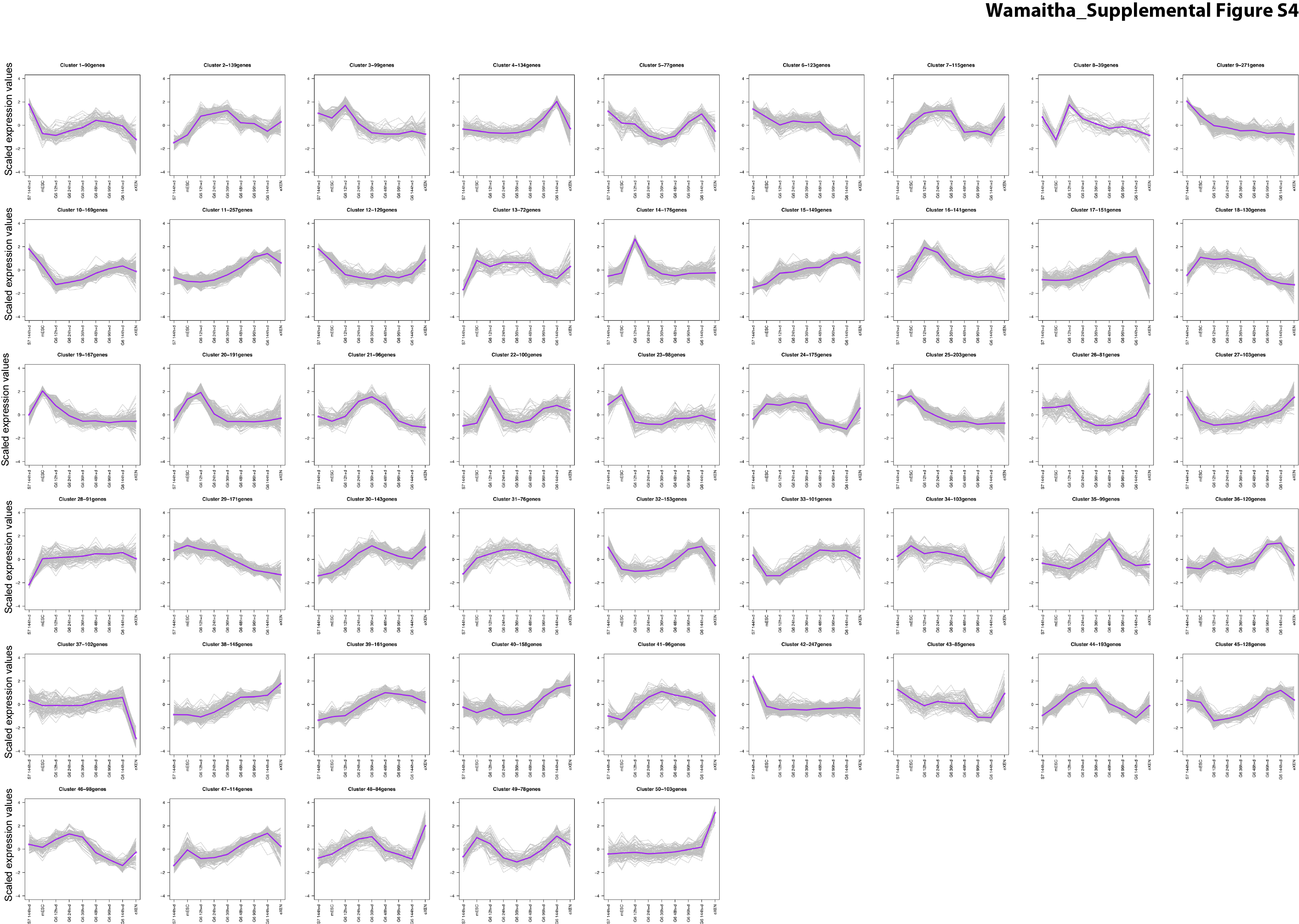

Supplement: Supplemental Material [file supp_29.12.1239_Supplemental_Figure_S4.tif]

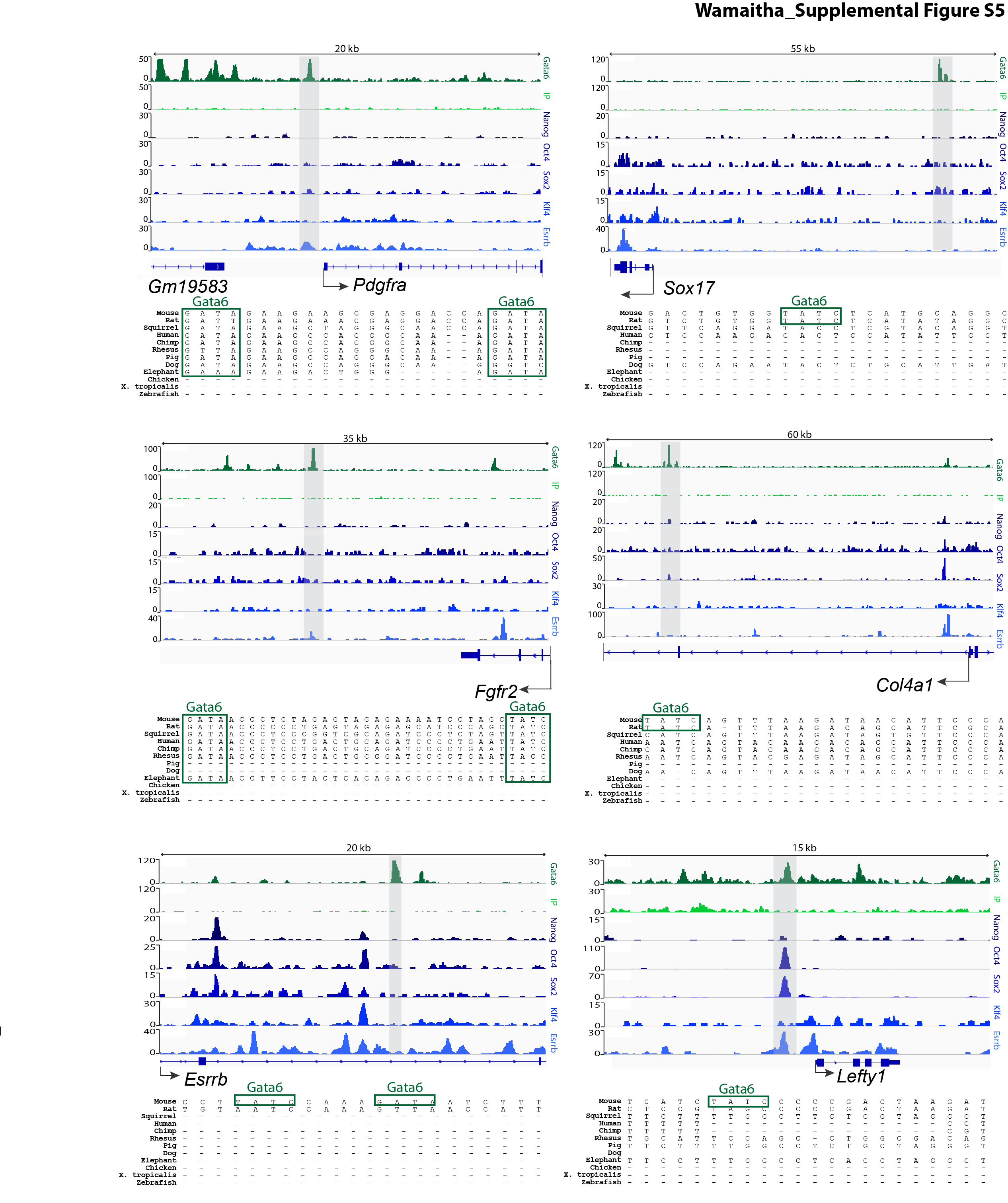

Supplement: Supplemental Material [file supp_29.12.1239_Supplemental_Figure_S5.tif]

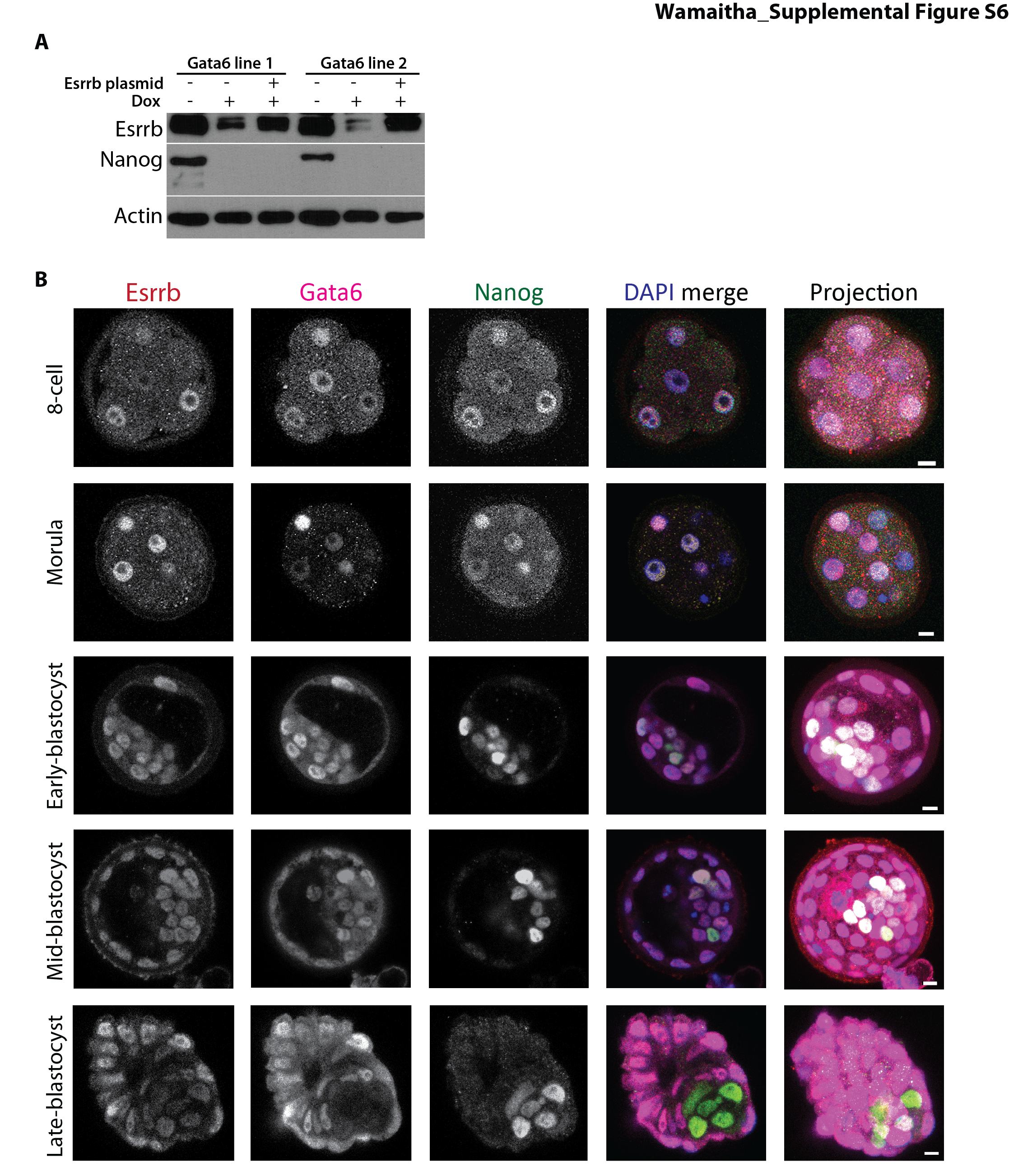

Supplement: Supplemental Material [file supp_29.12.1239_Supplemental_Figure_S6.tif]
