# Supplementary material for: Gata6 potently initiates reprograming of pluripotent and differentiated cells to extraembryonic endoderm stem cells
Source: Genes Dev. 2015 Jun 15;29(12):1239–55. doi: 10.1101/gad.257071.114 (PMC4495396; doi:10.1101/gad.257071.114)
Supplement: Supplemental Material [file supp_29.12.1239_SuppMaterial.docx]

**Gata6 potently initiates reprogramming of pluripotent and differentiated cells to extraembryonic endoderm stem cells**

**Sissy E. Wamaitha, Ignacio del Valle, Lily T. Y. Cho, Yingying Wei, Norah M. E. Fogarty, Paul Blakeley, Richard I. Sherwood, Hongkai Ji, Kathy K. Niakan**

**Inventory of Supplemental Information**

**1. Supplemental Figures**

Figure S1, related to Figure 1

Figure S2, related to Figures 2 and 3

Figure S3, related to Figure 2

Figure S4, related to Figure 3

Figure S5 related to Figure 4

Figure S6 related to Figure 5

Separate image files are provided for Figures S1 – S6. Corresponding figure legends are included in this document.

**2. Supplemental Experimental Procedures**

**3. Supplemental References**

**4. Supplemental Tables**

Table S1. Microarray analysis of Gata6 overexpressing cells

Table S2. ChIP-Seq analysis of Gata6 binding after 36 hours of doxycycline treatment (biologically triplicate cell lines)

Table S3. Gene ontology analysis of Gata6 bound genes

Table S4. Gene target overlap between Gata6-FLAG (36h) versus Gata6 (eXEN), Oct4, Sox2, Nanog, Klf4, Esrrb or Prdm14

Table S5. ChIP-Seq analysis of Gata6 binding in eXEN cell line 1

Table S6. ChIP-Seq analysis of Gata6 binding in eXEN cell line 2

Table S7. RNA-Seq analysis of GATA6 induction in hES cells (day 6) and control hES cells in KSR or mTeSR media

Table S8. DESeq analysis comparing hES cells in the presence or absence of Gata6 induction (day 6)

Table S9. KSR medium components

Table S10. Vector construction and genotyping primers

Table S11. Quantitative RT-PCR primers

Table S12. Antibodies used for immunohistochemistry, western blot and chromatin immunoprecipitation

Separate Excel files are provided for Tables S1 – S8. Corresponding table legends are included in this document.

**Supplemental Figures**

**Supplemental Figure S1. *Gata6* or *Gata4* induction is uniquely sufficient to reprogram mES cells to XEN cells.** (A) qRT-PCR analysis of *FLAG* expression (*Gata4, Gata6, Sox7* or *Sox17* transgene overexpressing mES cells) or V5 expression (*Foxa3* and *Hnf4a* transgene overexpressing mES cells) after 6 days of doxycycline treatment or *Gata4, Gata6, Sox7*, *Sox17* *Foxa3* and *Hnf4a* expression in embryo-derived XEN (eXEN). Induced cells were cultured in the presence or absence of LIF, or in the absence of LIF and supplemented with FGF and heparin, as indicated. Relative expression reflected as fold difference over wild-type mES cells normalized to *Gapdh*. Data are mean ± s.e.m. of 2-3 biological replicates and 4 technical replicates. (B) Immunofluorescence analysis for dsRed (red) with DAPI merge (blue) in untreated *dsRed* transgenic mES cells (upper panels) and after 2 days of doxycycline treatment (lower panels). Scale bars: 100 μm. (C) and (D) qRT-PCR analysis for selected pluripotency and endoderm transcripts in *dsRed, Foxa3, Hnf4a, Gata4, Gata6, Sox7* or *Sox17* induced cells after 6 days of doxycycline treatment. Cells were cultured in the presence or absence of LIF, or in the absence of LIF and supplemented with FGF and heparin, as indicated. Relative expression reflected as fold difference over uninduced mES cells normalized to *Gapdh*. Data are mean ± s.e.m. of 2-3 biological replicates and 4 technical replicates. *p<0.05, **p<0.01, ***p<0.001. (E) Representative phase contrast images of *dsRed, Foxa3, Hnf4a, Gata4, Gata6, Sox7* or *Sox17* induced cells after 6 days of doxycycline treatment in the absence of LIF and supplemented with FGF and heparin. Scale bars: 100 μm.

**Supplemental Figure S2. *Gata6* induction in wild-type and *Fgf4^-/-^* mES cells.** (A) Western blot for selected proteins in *Gata6* overexpressing cells from 0 to 12 hours of doxycycline induction. A representative Actin loading control is included as a reference. (B) qRT-PCR analysis for selected pluripotency and endoderm transcripts in late-passage self-renewing stable iXEN cells maintained for several months in the absence of exogenous Gata6 expression and embryo-derive XEN (eXEN) cells. (C) Immunofluorescence analysis of E3.5-4 chimera blastocysts for the expression of the primitive endoderm marker Sox17 (red), the epiblast marker Nanog (white), EGFP (green) and DAPI (blue), to determine chimera contribution of unlabeled Gata6-induced cells injected into B5/EGFP embryos that constitutively express EGFP. Scale bars: 100 μm. (D) Representative phase-contrast images of mES cells 6 days following transduction with a lentivirus driving exogenous expression of HA-tagged human GATA6 compared to uninduced cells. (E-G) qRT-PCR analysis for selected pluripotency and endoderm transcripts in (E) mES cells overexpressing HA-tagged human GATA6; (F) *Gata6* overexpressing Fgf4^-/-^ mES cells between 0 and 144 hours of doxycycline induction; or (G) late-passage Fgf4-/- iXEN cells maintained for several passages in the absence of exogenous Gata6. Data are mean ± s.e.m. of 2-3 biological replicates.

**Supplemental Figure S3. Flow cytometry analysis of *Gata6* induced pulse and pulse-chase cells.** FACS profiles for *Gata6* induced pulse and pulse-chase cells after immunostaining for Nanog expression (FITC) following 0 to 24 hours of doxycycline treatment (and subsequent 48 hour chase in pluripotency medium for pulse-chase samples). (A) and (C) Forward scatter (FSC) versus side scatter (SSC) plots of *Gata6* induced cells with putative population gating (All, P1 and P2). (B) and (D) Nanog-FITC versus SSC plots following doublet exclusion (data not shown). Gated populations are highlighted: All – red; P1 – blue; P2 – orange.

**Supplemental Figure S4. *Gata6* induction results in dynamic changes in gene expression.** Line plots generated following microarray analysis of *Gata6* induced cells from 12 to 144 hours following doxycycline treatment. Uninduced mES cells, embryo-derived XEN cells and *Sox7* overexpressing cells after 144 hours of doxycycline treatment were also included. Genes were grouped into 50 clusters using K-means clustering, according to normalized gene expression values scaled across time points. The trajectory of scaled expression values for individual genes in each cluster across time are shown as grey lines, while the purple lines correspond to the cluster mean.

**Supplemental Figure S5. Gata6 binds to known extraembryonic endoderm associated genes.** ChIP-seq binding profiles for *Pdgfra, Sox17, Fgfr2*, *Col4a1*, *Klf4, Lefty1, Nr5a2 and Esrrb* for Gata6 and input control during reprogramming compared to Nanog, Oct4, Sox2, Klf4 and Esrrb binding profiles in mES cells obtained from the Mouse ES Cell ChIP-Seq Compendium (Martello et al., 2012). Grey bar highlights co-occupancy or lack there-of. A cross-species comparison of a subset of the area within the grey bar was generated using the UCSC genome browser and the conserved GATA motif is highlighted.

**Supplemental Figure S6. *Esrrb* overexpression in *Gata6*-induced cells and expression in mouse preimplantation embryos.** (A) Western blot for selected proteins in *Esrrb* overexpressing *Gata6*-induced cells. Representative Actin loading controls are included as a reference. (B) Confocal optical sections and 3D projections of 8-cell, morula, early-, mid- and late-mouse blastocysts. Embryos were immunofluroescently analysed for the expression of Esrrb (red), Gata6 (magenta) and Nanog (green) with DAPI (blue) merge. Scale bar: 10 μm.

**Supplemental Experimental Procedures**

**Generation of inducible mES cells**

Inducible cell lines were generated as described in Beard et al., 2006 and Hochedlinger et al., 2005. We obtained KH2 mES cells that had been modified to include an M2-rtTA tetracycline-responsive transactivator under the control of the Rosa26 promoter, coupled with a tetracycline operator minimal promoter incorporated into the *Col1a* locus (Beard et al., 2006; Hochedlinger et al., 2005). *Gata4, Gata6, Hnf4a, Foxa3, Sox7* and *Sox17* cDNA sequences were amplified from XEN cell lines then integrated into a pgkATGfrt vector, which was co-electroporated with a pCAGGS-FLPe vector. Successfully targeted colonies were selected using Puromycin (1 μg/ml) and Hygromycin (50-100 μg/mL). For each of the ectopically expressed genes, three independent cell lines were clonally expanded and used as biological replicates. Additional details of the genotyping primer sequences can be found in Table S10.

To replicate this system in our *Fgf4-/-* mES cells we integrated the M2-rtTA tetracycline-responsive transactivator into the endogenous Rosa26 locus followed by targeting with the frt-neomycin/hygromycin-pA vector into the *Col1A* locus. Targeted cells were selected with Puromycin (1 μg/ml) and Neomycin (200 μg/mL). The double targeted *Fgf4-/-* cells were then electroporated with a vector carrying the Gata6 cDNA together with a pgkATG to restore Hygromycine resistance plus a pCAGGS-FLPe transient vector to facilitate recombination.

**Generation of inducible mES cells**

Inducible cell lines were generated as described in Beard et al., 2006 and Hochedlinger et al., 2005. We obtained KH2 mES cells that had been modified to include an M2-rtTA tetracycline-responsive transactivator under the control of the Rosa26 promoter, coupled with a tetracycline operator minimal promoter incorporated into the *Col1a* locus (Beard et al., 2006; Hochedlinger et al., 2005). *Gata4, Gata6, Hnf4a, Foxa3, Sox7* and *Sox17* cDNA sequences were amplified from XEN cell lines then integrated into a pgkATGfrt vector, which was co-electroporated with a pCAGGS-FLPe vector. Successfully targeted colonies were selected using Puromycin (1 μg/ml) and Hygromycin (50-100 μg/mL). For each of the ectopically expressed genes, three independent cell lines were clonally expanded and used as biological replicates. Additional details of the genotyping primer sequences can be found in Table S10.

To replicate this system in our *Fgf4-/-* mES cells we integrated the M2-rtTA tetracycline-responsive transactivator into the endogenous Rosa26 locus followed by targeting with the frt-neomycin/hygromycin-pA vector into the *Col1A* locus. Targeted cells were selected with Puromycin (1 μg/ml) and Neomycin (200 μg/mL). The double targeted *Fgf4-/-* cells were then electroporated with a vector carrying the Gata6 cDNA together with a pgkATG to restore Hygromycine resistance plus a pCAGGS-FLPe transient vector to facilitate recombination.

For experiments in the absence of exogenous FGF, *Fgf4^-/-^* Gata6-inducible cells were cultured for 3 or more passages on gelatin in serum-free neurobasal medium (NDiff 227, Cellartis/Takara Bio) supplemented with Erk inhibitor PD0325901 (Tocris) and Gsk3 inhibitor CHIR99021 (Axon), as for 2i+LIF conditions previously described (Ying et al., 2008). Doxycycline induction was carried out in serum-free neurobasal medium on gelatine in the absence of the 2 inhibitors.

**Flow cytometry**

Cells were dissociated using 0.05% Trypsin, then washed twice and resuspended in FACS fixing buffer (1% paraformaldehyde, 0.01% Tween in PBS) for 20 minutes on ice. Cells were washed twice with permeabilisation buffer (5% FBS, 0.05% Tween in PBS) then incubated with Nanog primary antibody (REC-RCAB0001P, 2B Scientific) and FITC anti-rabbit secondary antibody (A21206, Alexa Fluor) at a 1:250 dilution in permeabilisation buffer for 20 minutes on ice. Cells were washed twice then resuspended in permeabilisation buffer for analysis. Cells were analyzed on a BD Fortessa X20 flow cytometer (BD Biosciences). FlowJo software (BD Biosciences) was used to generate histogram overlays and dot plots.

**Chimera embryo generation and analysis**

Cells were injected into the blastocoel cavity of E2.5 – E3.5 B5/EGFP blastocysts, which constitutively express enhanced green fluorescent protein (EGFP) from a CMV early enhancer/chicken beta actin (CAG) promoter (Hadjantonakis et al., 1998). To investigate contribution to extraembryonic endoderm-derived tissues, 3 to 5 cells were injected per embryo and embryos transferred to the uterus of E2.5 vas plugged females. Dissections were performed between E5.5 and E6.5. To investigate contribution to the primitive endoderm, single cells were injected into each embryo. Embryos were cultured in KSOM mouse embryo culture medium and collected at E4.5. For further analysis, embryos were fixed in 4% paraformaldehyde for 1 hour on ice then prepared for immunohistochemistry as detailed below.

**Immunofluorescence analysis of mouse embryos**

6 weeks-old female Parkes or B5/EGFP mice were naturally mated. After detection of vaginal plug (E0.5) females were sacrificed and oviducts were dissected and removed to a petri-dish containing drops of M2 media at room temperature. The ampulla containing the zygotes was pierced and the clutch of zygotes was released into a drop of hyaluronidase solution for a few minutes to remove the cumulus cells. Zygotes were collected and washed through several drops of M2 media, before being transferred to a microdrop culture plate containing equilibrated KSOM media overlaid with mineral oil. Zygotes were washed through several drops of KSOM media and cultured at 37°C 5% CO_2_ to specific stages of development.

Embryos were collected and fixed in 4% paraformaldehyde at 4°C for 1 hour, permeabilized with 0.5% Tween in PBS for 20 minutes and blocked with 10% FBS diluted in 0.1% Tween in PBS for 1 hour. Primary antibodies Esrrb, Gata6, Sox17 or Nanog (Table S12) were diluted at 1:500 in blocking solution and samples incubated at 4°C rotating overnight. Alexa Fluor secondary antibodies (Molecular Probes, Life Technologies) were diluted at 1:300 in blocking solution and samples were incubated for 1 hour at room temperature, then washed and covered with 0.1% Tween in PBS containing DAPI Vectashield mounting medium (Vector Labs). Embryos were imaged on a Leica SP5 inverted confocal microscope (LeicaMicrosystems (UK) Ltd).

***Esrrb* overexpression**

Gata6 inducible mES cells were plated on gelatin-coated plates 24 hours prior to transfection so that the cells were approximately 70% confluent at the time of transduction. pCAGIP-Myc-Esrrb (Uranishi et al., 2013), PpyCAG-FLAG-Esrrb (van den Berg et al., 2008) and/or the pCAGGS-GFP plasmid were transfected into mES cells using Xfect reagent (Clontech) following the manufacturer’s instructions. Cells were supplemented with 1 μg/mL doxycycline at the time of transfection. Cells transfected only with the pCAGGS-GFP plasmid in the presence or absence of doxycycline were used as a control. Samples were collected and analysed 24 h after transfection.

**Neural stem cell lines**

The NS5 neural stem cell line (Conti et al., 2005) were cultured in Euromed-N media supplemented with L-glutamine, laminin, N2, BSA, FGF2 and EGF (R&D or LifeTechnologies). Cells were routinely passaged using Accutase (LifeTechnologies).

**Doxycycline inducible overexpression of *GATA6* in human ES cells**

Constitutive inducible expression of human *GATA6* (hGATA6) was performed using the Lenti-X Tet-On 3G inducible expression system (Clontech) following the manufacturer’s protocol. Firstly, the HA-tagged hGATA6 coding sequence was sub-cloned into the pLVX-TRE3G vector in order to generate the pLVX-TRE3G-hGATA6-HA vector. Lentiviral supernatants were subsequently obtained after transfection of HEK 293T cells with either the pLVX-TRE3G-hGATA6-HA or the pLVX-Tet3G vector using the X-fect reagent (Clontech). Supernatants were finally concentrated using the Lenti-X Concentrator (Clontech). H9 hES cells were sequentially transduced with the pLVX-Tet3G and the pLVX-TRE3G-hGATA6-HA lentivirus. Briefly, H9 hES cells were plated on Matrigel (BD Biosciences) in mTeSR1 medium (Stem Cell Technologies) one day before lentivirus transduction. Cells around 70% confluent were first transduced with the pLVX-tet3G lentivirus followed by selection with G418 (250 μg/ml). Resistant cells were selected for at least 4 passages and then transduced with the pLVX-TRE3G-hGATA6-HA lentivirus. For the induction of human GATA6 expression, doxycycline was added to the mTeSR1 media at a concentration of 2 μg/ml 24 hours post-transduction together with puromycin (1 μg/ml).

**RNA sequencing of untransduced and *GATA6*-transduced hES cells**

RNA was isolated using TRI Reagent (Sigma) and DNaseI treated (Ambion). Libraries were prepared using TruSeq RNA Library Preparation Kit v2 (Illumina). Quality of total RNA and subsequent cDNA library preparations was assessed on a 2100 Bioanalyzer (Agilent). Libraries were submitted for 50-bp paired-end sequencing on an Illumina HiSeq 2000 (Illumina).

**Computational analysis of microarray data**

The raw data was pre-processed using the Bioconductor R package beadarray (Dunning et al., 2007). The raw expression values were first log2 transformed and then quantile normalized (Bolstad et al., 2003). Batch effects were corrected following the approach in (Johnson et al., 2007). Coefficient of variation (CV) was calculated for each probe across all samples according to the normalized values. To investigate patterns of expression changes over time, the normalized expression values were further scaled across time points. K-means clustering with k=50 was performed on the scaled expression data.

**Computational analysis of ChIP-seq data**

ChIP-seq data was assessed using the FASTQC program for quality (Babraham Bioinformatics). Any residual adapters were removed using Trim Galore! (Babraham Bioinformatics). Read were aligned to the UCSC mouse mm9 or mm10 reference genomes using Bowtie2 (Langmead and Salzberg, 2012) with the following parameters:

Gata6: bowtie2 -t -k 2 -N 1 --mm -x mm10 -1 sample.fastq -2 sample.fastq -S sample.sam

Only uniquely matched reads were retained using a sed command. Aligned reads (SAM files) were then transformed into a BED format and subsequently used to generate bigwig files. The bigwig files were visualized using the IGV browser (Robinson et al., 2011). Gata6 36-hour post doxycycline or eXEN cell replicates were compared using wigcorrelate and individual BAM files were merged. Candidate peaks were obtained using either CisGenome or MACS2 with an FDR <0.01. Peaks were called relative to input control samples. Gene annotation was performed using HOMER (Heinz et al., 2010). *De novo* motif analysis of the Gata6 ChIP-seq data was carried out using MEME (Bailey et al., 2009). The distribution of Gata6 binding relative to the mouse genome was analysed using CEAS (Shin et al., 2009).

Additional ChIP-seq datasets used in this study (Oct4, Sox2, Nanog, Esrrb, Suz12 and Ezh2) were obtained from the ES cell ChIP-seq compendium (Martello et al., 2012), accessible from:

<http://bioinformatics.cscr.cam.ac.uk/ES_Cell_ChIP-seq_compendium.html>.

BEDOPS was used to calculate the intersection between BED files (Neph et al., 2012). Heatmaps showing average ChIP enrichment relative to genomic regions were generated using deepTools (developed by the Bioinformatics Facility at the Max Planck Institute for Immunobiology and Epigenetics, Freiburg). Venn diagrams of genes corresponding to different datasets were performed using BioVenn (Hulsen et al., 2008).

**Computational analysis of RNA-seq data**

Paired-end reads were aligned to the human genome (hg19/GRCh37) using TopHat2 (Kim et al., 2013). BAM files were sorted and converted into SAM files using SAMtools (Li et al., 2009). The python package *HTSeq* (Anders et al., 2015) was then used to calculate read counts for each ensemble gene model (release 74), and these values were normalized by both gene length and sequencing depth to generate RPKM expression values. A heatmap representing hierarchical clustering of pluripotency and endoderm associated gene was generated, based on scaled RPKM values using the R package *pheatmap*. Principal components analysis was performed using the prcomp function in the R *stats* package based on RPKM expression values. The resulting count files for each sample were also used as input for differential expression analysis using DESeq.

**Supplemental References**

Anders, S., Pyl, P.T., and Huber, W. (2015). HTSeq--a Python framework to work with high-throughput sequencing data. Bioinformatics *31*, 166-169.

Bailey, T.L., Boden, M., Buske, F.A., Frith, M., Grant, C.E., Clementi, L., Ren, J., Li, W.W., and Noble, W.S. (2009). MEME SUITE: tools for motif discovery and searching. Nucleic acids research *37*, W202-208.

Beard, C., Hochedlinger, K., Plath, K., Wutz, A., and Jaenisch, R. (2006). Efficient method to generate single-copy transgenic mice by site-specific integration in embryonic stem cells. Genesis *44*, 23-28.

Bolstad, B.M., Irizarry, R.A., Astrand, M., and Speed, T.P. (2003). A comparison of normalization methods for high density oligonucleotide array data based on variance and bias. Bioinformatics *19*, 185-193.

Conti, L., Pollard, S.M., Gorba, T., Reitano, E., Toselli, M., Biella, G., Sun, Y., Sanzone, S., Ying, Q.L., Cattaneo, E.*, et al.* (2005). Niche-independent symmetrical self-renewal of a mammalian tissue stem cell. PLoS biology *3*, e283.

Dunning, M.J., Smith, M.L., Ritchie, M.E., and Tavare, S. (2007). beadarray: R classes and methods for Illumina bead-based data. Bioinformatics *23*, 2183-2184.

Heinz, S., Benner, C., Spann, N., Bertolino, E., Lin, Y.C., Laslo, P., Cheng, J.X., Murre, C., Singh, H., and Glass, C.K. (2010). Simple combinations of lineage-determining transcription factors prime cis-regulatory elements required for macrophage and B cell identities. Molecular cell *38*, 576-589.

Hochedlinger, K., Yamada, Y., Beard, C., and Jaenisch, R. (2005). Ectopic expression of Oct-4 blocks progenitor-cell differentiation and causes dysplasia in epithelial tissues. Cell *121*, 465-477.

Hulsen, T., de Vlieg, J., and Alkema, W. (2008). BioVenn - a web application for the comparison and visualization of biological lists using area-proportional Venn diagrams. BMC genomics *9*, 488.

Johnson, W.E., Li, C., and Rabinovic, A. (2007). Adjusting batch effects in microarray expression data using empirical Bayes methods. Biostatistics *8*, 118-127.

Kim, D., Pertea, G., Trapnell, C., Pimentel, H., Kelley, R., and Salzberg, S.L. (2013). TopHat2: accurate alignment of transcriptomes in the presence of insertions, deletions and gene fusions. Genome biology *14*, R36.

Langmead, B., and Salzberg, S.L. (2012). Fast gapped-read alignment with Bowtie 2. Nature methods *9*, 357-359.

Li, H., Handsaker, B., Wysoker, A., Fennell, T., Ruan, J., Homer, N., Marth, G., Abecasis, G., Durbin, R., and Genome Project Data Processing, S. (2009). The Sequence Alignment/Map format and SAMtools. Bioinformatics *25*, 2078-2079.

Martello, G., Sugimoto, T., Diamanti, E., Joshi, A., Hannah, R., Ohtsuka, S., Gottgens, B., Niwa, H., and Smith, A. (2012). Esrrb is a pivotal target of the Gsk3/Tcf3 axis regulating embryonic stem cell self-renewal. Cell stem cell *11*, 491-504.

Neph, S., Kuehn, M.S., Reynolds, A.P., Haugen, E., Thurman, R.E., Johnson, A.K., Rynes, E., Maurano, M.T., Vierstra, J., Thomas, S.*, et al.* (2012). BEDOPS: high-performance genomic feature operations. Bioinformatics *28*, 1919-1920.

Robinson, J.T., Thorvaldsdottir, H., Winckler, W., Guttman, M., Lander, E.S., Getz, G., and Mesirov, J.P. (2011). Integrative genomics viewer. Nature biotechnology *29*, 24-26.

Shin, H., Liu, T., Manrai, A.K., and Liu, X.S. (2009). CEAS: cis-regulatory element annotation system. Bioinformatics *25*, 2605-2606.

Uranishi, K., Akagi, T., Sun, C., Koide, H., and Yokota, T. (2013). Dax1 associates with Esrrb and regulates its function in embryonic stem cells. Molecular and cellular biology *33*, 2056-2066.

van den Berg, D.L., Zhang, W., Yates, A., Engelen, E., Takacs, K., Bezstarosti, K., Demmers, J., Chambers, I., and Poot, R.A. (2008). Estrogen-related receptor beta interacts with Oct4 to positively regulate Nanog gene expression. Molecular and cellular biology *28*, 5986-5995.

Ying, Q.L., Wray, J., Nichols, J., Batlle-Morera, L., Doble, B., Woodgett, J., Cohen, P., and Smith, A. (2008). The ground state of embryonic stem cell self-renewal. Nature *453*, 519-523.

**Supplemental Tables**

**Table S1. Microarray analysis of Gata6 overexpressing cells at defined timepoints between 12 and 144 hours of doxycycline treatment.**

Differentially expressed genes are listed alphabetically within their respective clusters. Uninduced mES cells, embryo-derived XEN cells and *Sox7* overexpressing cells after 144 hours of doxycycline treatment were also included.

**Table S2. ChIP-Seq analysis of Gata6 binding after 36 hours of doxycycline treatment.**

Gata6 bound regions are listed according to their false discovery rate.

**Table S3. Gene ontology analysis of Gata6-bound dynamically expressed genes.**

**Table S4. Comparison of overlapping gene targets between Gata6 and pluripotency-associated factors.**

Comparison of Gata6 target genes during reprogramming compared to Oct4, Sox2, Nanog, Klf4 or Esrrb target genes in mES cells. (B) Comparison of Sox17 bound genes compared to Oct4, Sox2, Nanog, Klf4 or Esrrb bound genes in mES cells. (C) Gata6 target genes during reprogramming compared to Prdm14 and Ring1B target genes. Data for Oct4, Sox2, Nanog, Klf4, Esrrb and Prdm14 was obtained from the Mouse ES Cell ChIP-Seq Compendium (Martello et al., 2012), Ring1b from (Brookes et al., 2012) and Sox17 from (Niakan et al., 2010).

**Table S5. ChIP-Seq analysis of Gata6 binding in embryo-derived XEN cell line 2.**

Gata6 bound regions with a false discovery rate less than 0.01 are listed.

**Table S6. ChIP-Seq analysis of Gata6 binding in embryo-derived XEN cell line 1.**

Gata6 bound regions with a false discovery rate less than 0.01 are listed.

**Table S7: RNAseq analysis of untransduced control and *GATA6*-induced hES cells.**

RNA sequencing analysis for two technical replicates per condition for untransduced control hES cells (KSR1, KSR2, mTESR1 mTeSR2) and *GATA6*-transduced hES cells (+GATA6 KSR1, +GATA6 KSR2, +GATA6 mTeSR1, +GATA6 mTeSR2) after 6 days in KSR or mTeSR media.

**Table S8. DESeq analysis comparing differential gene expression in untransduced and *GATA6*-transduced hES cells**

Separate Excel files are provided for Supplemental Tables S1-S8,

**Table S9. Components for Knockout Serum Replacer medium (KSR Medium) (500 ml)**

| **Component** | **Supplier** | **Catalogue Number** | **Stock Concentration** | **Final concentration** | **Volume Used** |
| --- | --- | --- | --- | --- | --- |
| Advanced DMEM/F12 | Invitrogen | 12634010 | - | 78% | 390 ml |
| Knockout serum replacer | Invitrogen | 10828028 | - | 20% | 100 ml |
| L-glutamine | Sigma | 25030024 | 200 mM | 2 mM | 5 ml |
| β mercaptoethanol | Sigma | M6250 | 14.3 M | 0.1 mM | 3.5 μl |
| \| Penicillin/  Streptomycin \| \| --- \| | Sigma | 15140-122 | 100% | 1% | 5 ml |

#### Table S10. Vector construction and genotyping primers

| Name | **Forward Primer** | **Reverse Primer** |
| --- | --- | --- |
| Detecting M2RTTA 5’ incorporation | LTC_R26_5’M2RTTA_GT_spanXBA_F  CTTTCTAGAATTCGTCGATCG | LTC_R26_5’M2RTTA_GT_own design R2  GAATGACTTGGCGTTGTTCC |
| Detecting M2RTTA 3’ incorporation | LTC_R26_3’M2RTTA_GT_spanXBA_F  TCTAGACTTAGAACCACAAAG | LTC_R26_5’M2RTTA_GT_own design R1  GCAACCTCCCCTTCTACGA |
| Detecting Col1a incorporation | Col1a/Frt Forward primer  GAAGTTCCTATTCCGAAGTTC | TetO Reverse primer (on Gata6 cDNA vector)  TATAGGCCTCCCACCGTACA |
| Gata6 cDNA incorporation | GACCGACGCGTCACCATGGCCTTGACTGACGGC | GACCGACGCGTGGCCAGGGCCAGAGC |
| Gata4 cDNA incorporation | GACCGACGCGTCACCATGTACCAAAGCCTGGCC | GACCGACGCGTCGCGGTGATTATGTCCCC |
| Sox7 cDNA incorporation | GACCGACGCGTCACCATGGCCTCGCTGCTGGGC | GACCGACGCGTTGACACACTGTAGCT |
| 31'sequencing primer | TCGTTTAGTGAACCGTCAGA | CATCGTCATCCTTGTAGTCG |
| 31'RFP sequencing primer | TACTTGTCATCGTCATCCTTG | GTACCCGGGGATCCTCTA |

#### Table S11. Quantitative RT-PCR primers

| Mouse quantitative RT-PCR primers | | |
| --- | --- | --- |
| **Name** | **Forward Primer** | **Reverse Primer** |
| *Col4a1* | GGTCCTGTCTGGAAGAGTTT | AAATACAATGGGAGGGAGAA |
| *Dab2* | GGCAACAGGCTGAACCATTAGT | TTGGTGTCGATTTCAGAGTTTAGAT |
| *Esrrb 3’UTR* | CCTCCACTTTCCCCTTTCTT | AGGTTTCTAGACTGGCCATG |
| *FLAG* | CAAAGACCATGACGGTGATTA | TCGTCATCCTTGTAGTCGATG |
| *Foxa3 3’UTR* | GGCTGTGTGCCTCAGGTCGG | CTCCGGTGCTGCCACCAGTG |
| *Gapdh* | AATGGAATACGGCTACAGC | GTGCAGCGAACTTTATTG |
| *Gata4 3’UTR* | TTCCTGCTCGGACTTGGGAC | TGGACAGGCAGGTGGAGAATAAG |
| *Gata6 3’UTR* | ACAGCCCACTTCTGTGTTCCC | GTGGGTTGGTCACGTGGTACAG |
| *V5* | CGGTTCGAAGGTAAGCCTAT | ATGGTGATGGTGATGATGA |
| *Hnf4a 3’UTR* | CAACGATCACCAAGCAAGAA | CTGGGGTCTTCTCAAGCAAA |
| *Lama1* | AGGTCTGCGTTGAGTGTTCTG | CAGTACTATGCCGTCAGCGAT |
| *Nanog* | AGCAGATGCAAGAACTCTCCTC | AAGTTGGGTTGGTCCAAGTCT |
| *Pou5f1* | AGAAGTCCCAGGACATCAAA | TGCTTTGCATATCTCCTGAA |
| *Prdm14 3’UTR* | ATTTGGTTTTGGTGGTGGTG | CCTGGCATTTTCATTGCTCA |
| *Sox2* | TAGAGCTAGACTCCGGGCGATGA | TTGCCTTAAACAAGACCACGAAA |
| *Sox7 3’UTR* | AACACTCCCGGCCACCCTGA | AAGGAGCGCTGGATCCTGGCT |
| *Sox17 3’UTR* | AAGAAACCCTAAACACAAACAGCG | TTTGTGGGAAGTGGGATCAAGAC |
| *Sparc1* | AGGGCCTGGATCTTCTTTCTC | CAAATTCTCCCATTTCCACCT |
| Human quantitative RT-PCR primers | | |
| *AFP* | ACCATGAAGTGGGTGGAATC | TGGTAGCCAGGTCAGCTAAA |
| *GATA6* | TCCACTCGTGTCTGCTTTTG | TCCTAGTCCTGGCTTCTGGA |
| *HA-GATA6* | AACACAACCTACAGCCTCAG | GACGTCATAAGGGTAGGCC |
| *NANOG* | GCAACCTGAAGACGTGTGAA | CTCGCTGATTAGGCTCCAAC |
| *POU5F1* | TATGGGAGCCCTCACTTCAC | CAAAAACCCTGGCACAAACT |
| *SOX2* | TTGTTCGATCCCAACTTTCC | ACATGGATTCTCGGCAGACT |
| *SOX7* | ACGCCGAGCTCAGCAAGAT | TCCACGTACGGCCTCTTCTG |
| *SOX17* | CGCACGGAATTTGAACAGTA | GGATCAGGGACCTGTCACAC |

**Table S12. Antibodies used for immunohistochemistry (IHC), western blot (WB) and chromatin immunoprecipitation (ChIP)**

| **Primary**  **Antibody** | **Supplier** | **Catalogue Number** | **Application** | **Species** | **Dilution used** |
| --- | --- | --- | --- | --- | --- |
| Actin | Millipore | MAB1501 | WB | Mouse | 1:10000 |
| Esrrb | R&D | PP-H6705-00 | IHC | Mouse | 1:500 |
| FLAG | Sigma | F1804 | IHC/WB/ChIP | Mouse | 1:500/1:1000/9μg per mL |
| Gata4 | Santa Cruz Biotech | SC-9053 | IHC/WB | Rabbit | 1:500/1:1000 |
| Gata6 | R&D | AF1700 | IHC/WB | Goat | 1:500/1:1000  Mouse |
| Gata6 | Santa Cruz Biotech | SC-9055 | IHC | Rabbit | 1:500  Human |
| HA | Roche | 11867423001 | IHC | Rat | 1:500 |
| p44/42 MAPK (Erk1/2) | Cell Signaling | 4695 | WB | Rabbit | 1:2000 |
| Phospho p44/42 MAPK | Cell Signaling | 9101 | WB | Rabbit | 1:1000 |
| Nanog | 2B Scientific | REC-RCAB0001P | IHC/WB | Rabbit | 1:1000  Mouse |
| Nanog | R&D | AF1977 | IHC | Goat | 1:500  Human |
| Oct4 | Santa Cruz Biotech | SC-5279 | IHC/WB | Mouse | 1:500/1:1000 |
| Sox2 | R&D | AF2018 | WB | Goat | 1:1000 |
| Sox7 | R&D | MAB2766 | IHC | Goat | 1:500 |
| Sox17 | R&D | AF1924 | IHC/WB | Goat | 1:500/1:1000 |
|  |  |  |  |  |  |
| **Secondary**  **Antibody** | **Supplier** | **Catalogue No.** | **Application** | **Species** | **Dilution** |
| Alexa Fluor  anti-mouse IgG | Invitrogen | A21202 (488)  A21203 (594)  A31571 (647) | IHC | Donkey | 1:300 |
| Alexa Fluor  anti-rabbit IgG | Invitrogen | A21206 (488)  A21207 (594)  A31573 (647) | IHC | Donkey | 1:300 |
| Alexa Fluor  anti-goat IgG | Invitrogen | A11055 (488)  A11058 (594)  A21447 (647) | IHC | Donkey | 1:300 |
| Goat IgG (H+L),  HRP conjugated | Santa Cruz | SC-2020 | WB | Goat | 1:20000 |
| Mouse IgG (H+L), HRP conjugated | Cell Signaling | 7076 | WB | Mouse | 1:20000 |
| Rabbit IgG (H+L), HRP conjugated | Cell Signaling | 7074 | WB | Rabbit | 1:20000 |
